# The evolution of hierarchically structured communication

**DOI:** 10.3389/fpsyg.2023.1224324

**Published:** 2023-09-11

**Authors:** Ronald J. Planer

**Affiliations:** ^1^School of Liberal Arts, University of Wollongong, Wollongong, NSW, Australia; ^2^Words, Bones, Genes, and Tools: DFG Center for Advanced Studies, University of Tübingen, Tübingen, Germany

**Keywords:** hierarchical structure, hierarchical cognition, formal grammar, action structure, conversation, repair initiators, stone tools, copying

## Abstract

Human language sentences are standardly understood as exhibiting considerable hierarchical structure: they can and typically do contain parts that in turn contain parts, etc. In other words, sentences are thought to generally exhibit significant nested part-whole structure. As far as we can tell, this is not a feature of the gestural or vocal communication systems of our great ape relatives. So, one of the many challenges we face in providing a theory of human language evolution is to explain the evolution of hierarchically structured communication in our line. This article takes up that challenge. More specifically, I first present and motivate an account of hierarchical structure in language that departs significantly from the orthodox conception of such structure in linguistics and evolutionary discussions that draw on linguistic theory. On the account I propose, linguistic structure, including hierarchical structure, is treated as a special case of structured action. This account is rooted in the cognitive neuroscience of action, as opposed to (formal) linguistic theory. Among other things, such an account enables us to see how selection for enhanced capacities of act organization and act control in actors, and for act interpretation in observers, might have constructed the brain machinery necessary for the elaborate forms of hierarchically structured communication that we humans engage in. I flesh out this line of thought, emphasizing in particular the role of hominin technique and technology, and the social learning thereof, as evolutionary drivers of this brain machinery.

## 1. Introduction: hierarchical structure in language

It is often taken as a given that all but the simplest human language sentences are hierarchically structured. In other words, sentences are typically understood as exhibiting nested part-whole structure: they have parts that in turn have parts, etc. But what is it in virtue of which certain linguistic items making up a sentence count as forming a genuine *constituent* of that sentence—that is, a cohesive, (quasi-)independent chunk that might be moved around in the sentence, or substituted for another such linguistic entity—whereas others do not? As will become clear below, this question turns out to be a non-trivial one to answer. To start, though, let us just consider an example. Take (1):

(1) The boy with the broken leg missed the game.

**Figure 1:**
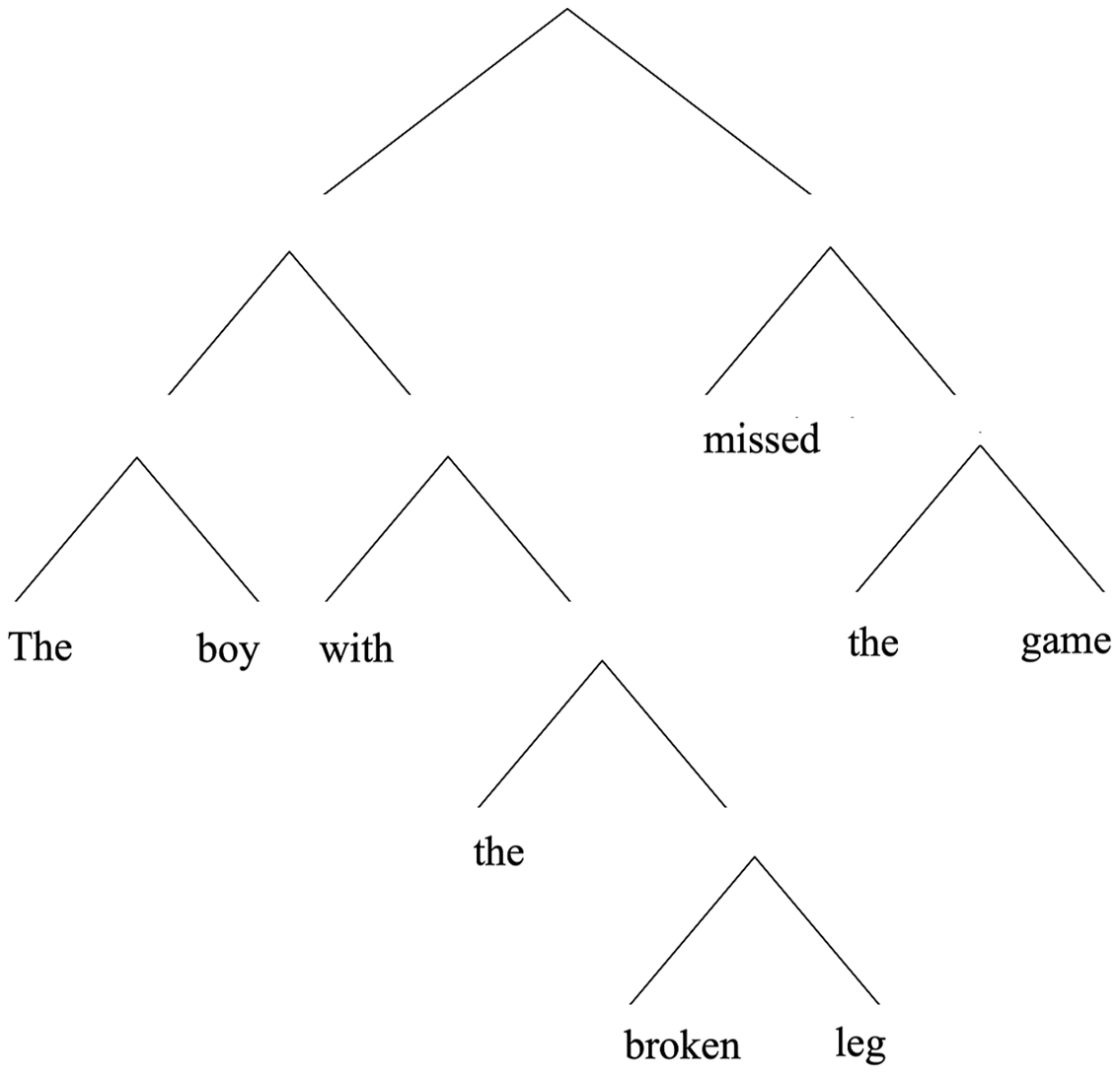
A hierarchical representation of (1).

Here, “the boy with the broken leg,” is intuitively understood as a constituent of (1), as is “with the broken leg” (as is “the broken leg” …). However, other ways of grouping together words—including words that form a contiguous unit—do not intuitively count as a legitimate constituent. The phrase “leg missed the” does not seem like a proper constituent of the sentence, for example. So, already we see that the idea of hierarchical structure in language is not simply that words in a sentence can be grouped together with adjacent words. Such intuitions about constituency are often represented with tree diagrams, as in Figure 1.

Hierarchical structure in this sense should be distinguished from a related but distinct type of structure known as *center embedding*. With center embedding, not only is it the case that one constituent occurs inside another; the former “splits up” the larger containing constituent, and in this sense occurs at (or near) the latter’s center. (2), below, is an extremely simple example:

(2) The boy the dog bit went to hospital.

**Figure 2:**
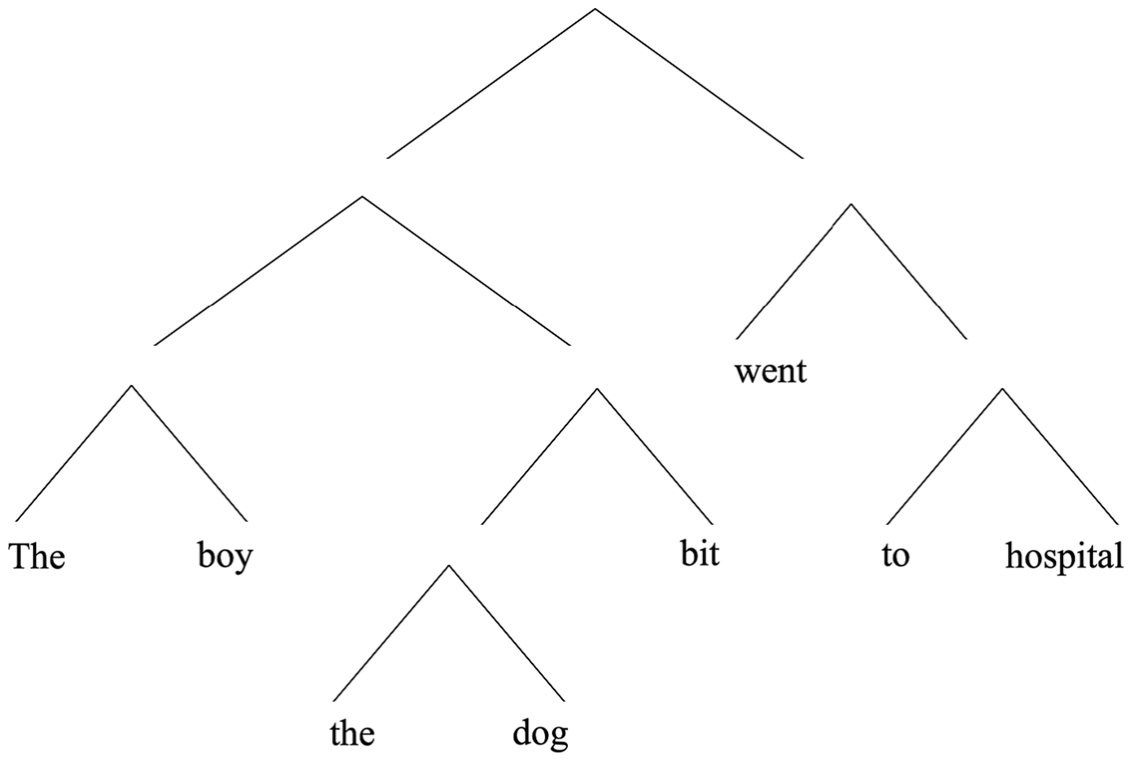
A hierarchical representation of (2).

In (2), “the boy the dog bit,” is intuitively a constituent, but so is “the dog.” Note that “the dog” is flanked on both sides by material belonging to the larger containing whole (see Figure 2). This contrasts with the way “with the broken leg,” is embedded in “the boy with the broken leg” in (1) (cf. Figure 1).

Center embedding thus brings about a *type* of nested part-whole structure, but a sentence can have nested part-whole structure (and hence count as hierarchically structured) without exhibiting center embedding—something (1) serves to clearly bring out. Despite this, hierarchical structure and center embedding have sometimes been treated, at least in practice, as a package deal (see e.g., Hauser et al., 2002; Fitch and Hauser, 2004; Gentner et al., 2006; Makuuchi et al., 2009).^1^ This is unfortunate, as skepticism (which may or may not be ultimately justified) regarding the presence/degree of the latter in human languages can unduly spread to the former. For example, Dan Everrett’s claims (see, in particular, Everett, 2005) that Pirahã shows no signs of embedding have tended to generate intense disagreement among linguists (see, e.g., Nevins et al., 2009 for a strong skeptical response).^2^

Whatever conclusions debates about center embedding in human language ultimately reach, and whatever broader significance winds up being attached to said conclusions, it’s clear that the issue of hierarchical structure *as such* in language is a distinct one. To see this, it helps to appreciate just how mundane cases of nested part-whole structure in language can be. Take (3), for example:

(3) The boy and the girl walked.

This sentence makes use of a syntactic device known as *noun-phrase coordination* (“NP coordination”). (3) contains a single verb, “walked,” which takes a single NP as an argument. What NP coordination does in English is allow speakers to combine (e.g., by using “and”) multiple NPs into a single constituent that is capable of filling a single argument slot. In this way, we English speakers can in principle build NPs of arbitrary complexity (e.g., “the boy and the girl and the cat and the dog … walked”). But—and this is the crucial point—NP coordination is obviously demanding of an ability to nest multiple lower-level parts into a larger cohesive whole, resulting in a form of nested part-whole structure at the sentence level.

Why think hierarchical structure is an interesting feature of human language? Well, as we just saw, it’s central to how we build up (ever more) complex linguistic structures. In his famous “The Architecture of Complexity” (Herbert, 1962), Herb Simon singled out hierarchical structure as a general design principle for building complexity in nature. He also plausibly suggested that hierarchical structure facilitates evolvability. Such structure simultaneously allows for both sweeping and surgical changes to a system’s organization. From this view, the extensive hierarchical structure of genomes is no surprise (see, e.g., Carroll, 2005; Planer, 2014). But as relates directly to the case of human language: hierarchical structure would appear to be absent from non-human great ape (hereafter, “great ape”) forms of communication. Great apes do sometimes produce complex gesture sequences; however, there’s no indication that the constituent gestures form cohesive subunits (Genty and Byrne, 2010; Byrne et al., 2017). Indeed, it seems that the signs belonging to a gesture stream do not even work together to form a complex meaning, much less a meaning that’s greater than the sum of the meanings of the individual parts.^3^ Instead, it appears that when great apes are unsure of which type of gesture is likely to be effective with a receiver, they simply “throw out” many gestures in a rapid-fire sequence, in the hopes that at least one will work. This explains why the number of gestures per gesture sequence tends to fall over time; senders learn which signs work (in a given context), and which do not. Eventually, the sequence is replaced by just a single sign.

Work by Truswell (2017) sheds light on the absence of hierarchically structured signs in great ape communication systems. Truswell reanalyzed Sue Savage-Rumbaugh’s corpus of her communicative interactions with the extensively enculturated male bonobo, Kanzi.^4^ Among other things, Truswell found a steep drop-off in Kanzi’s accuracy in complying with requests involving NP coordination, as compared to Kanzi’s accuracy across the whole corpus. (4) is an example of such a request that Kanzi received:

(4) Fetch the oil and the tomato.

As Truswell pointed out, in order to understand and hence reliably comply with a request of this sort, one must be able to represent the two NPs as forming a single constituent which serves as a single argument for the verb. Otherwise, one is left with an extra NP which plays no clear role in the sentence (e.g., “Fetch me the oil. The tomato.”). Thus, to the extent that Kanzi is unable to combine NPs in this way, his performance should suffer in such cases. More specifically, Truswell reasoned that: (i) a third of the time, Kanzi should ignore one of the NPs; (ii) a third of the time, Kanzi should ignore the other NP; and (iii) a third of the time, he should serendipitously fulfill the request.

Truswell identified 26 requests in Savage-Rumbaugh’s corpus featuring NP coordination. However, he discarded 8 due to various confounds (e.g., because Kanzi was already in contact with one of the two relevant objects at the time of request).^5^ Truswell found that: (i) in 9 of the remaining 18 cases, Kanzi ignored the first NP; (ii) in 5, Kanzi ignored the second NP; while (iii) in the final 4 cases, Kanzi accurately fulfilled the request. Kanzi’s performance was thus not far off Truswell’s prediction. Kanzi’s accuracy for requests involving NP coordination was a mere 22%, in contrast to his accuracy across the entire corpus, which Truswell estimated to be at 71.5%. While the sample size of sentences involving NP coordination is admittedly small, the contrast is certainly striking.^6^

Such evidence is suggestive of a significant difference in cognitive architecture between humans and other great apes in this area.^7^ Whereas humans have little to no difficulty communicating with a wide range of hierarchically structured utterances—including ones with rather “deep” levels of hierarchical structure—it seems that even very basic forms of hierarchical structure [e.g., (4)] pose significant difficulties for our great ape relatives. If so, then explaining the evolution of humans’ tremendous facility for communication involving hierarchically structured signs becomes a crucial—and perhaps an especially crucial—part of the challenge of explaining the evolution of humans’ linguistic abilities more generally.

My goal in this article is to sketch such an account. The account I shall develop closely follows an earlier account presented in Planer and Sterelny (2021). In particular, it shares that account’s novel treatment of the nature of linguistic structure, which includes hierarchical structure. In essence, linguistic structure is treated as a special case of action structure. This reframing of the nature of linguistic structure in turn reframes the evolutionary agenda (i.e., that which stands in need of an evolutionary explanation). Likewise, the present account gives a central role to the activities of stone tool manufacture and use in explaining the evolution of the cognitive machinery necessary for complex and fluent hierarchical communication. However, in addition to providing some additional details of import, the present account offers a different explanation of the evolution of our ability *as receivers* to hierarchically analyze others’ utterances. I explain why I now think such a change is necessary.

The article takes up these issues in the above order. But before getting on with this work package, let me explain why one might think a fresh approach to understanding the nature of linguistic structure is needed.^8^ Let us return to the question raised at the start of this article: exactly what is it that makes some but not other bits of language making up a sentence proper constituents? Why is it, for example, that “with the broken leg” counts as a genuine constituent of (1), whereas “leg missed the” does not? The latter is certainly a *physical* part of (1).

There is a received, albeit often implicit, answer to this question that runs something like this. Facts about linguistic constituents—and hence nested part-whole structure—are fixed by the *grammar* underlying the relevant language. By “grammar,” I here mean a system of abstract rules and symbols (both non-terminal and terminal)^9^ which together generate the set of possible (i.e., well-formed) sentences in the language. A *formal* grammar, in other words. So, the reason “with the broken leg” counts as a constituent of (1), whereas “leg missed the” does not, is that only the former is a legitimate way of filling out a language category posited by the grammar underlying English (in this case: a Prepositional Phrase, or PP).

On this view, then, structure is imposed on a sentence (a “string” in the formal linguistics jargon) by the formal grammar generating it. And the grammars underlying human languages have generally been assumed to belong to a special class of grammars known as *context-free grammars* (see, e.g., Chomsky, 1959; Charniak and McDermott, 1985; Haegeman, 1991). Here, we can keep the formal details to a minimum. Suffice it to say that the defining feature of a context-free grammar is that it can contain rules that allow a non-terminal symbol to be “rewritten” by two or more non-terminal symbols (e.g., A ➔ BC). This is precisely the feature that confers upon sentences nested part-whole structure; it’s what allows for structure to be built “from within.”^10^ A *regular grammar*, in contrast, cannot contain such rules; each rule in a regular grammar allows you to “rewrite” a non-terminal symbol on one side of a rule with at most a single non-terminal on the other (e.g., A ➔ B, or A ➔ Ba). The result is that you can only build structure from the edge of a string. A sentence cannot be expanded “from the inside out.”^11^

However, such an account of structure arguably raises more questions than it answers. First, we might ask, what justifies treating a certain grammar as *the* grammar underlying a given language—say, English? Speakers’ intuitions play a crucial role. As English speakers, we make certain intuitive judgments about what the various parts of sentences are, about how those sentences are structured. In turn, this motivates positing a certain grammar for the language—namely, one that generates (and only generates!) sentences that have this very structure. But is not it circular to turn around and then explain why certain physical parts of a sentence count as constituents by appeal to the language’s grammar, one might wonder? Perhaps not if grammars are psychologically real entities, that is, actual computational systems we carry in our heads and which we activate in producing and comprehending sentences. This was certainly the idea in the early days of generative linguistics. And it’s easy to see why such an idea was attractive: our brains are finite physical systems, which nonetheless manifest an infinite linguistic capacity; a formal grammar elegantly explains how such a thing can be possible. However, by as early as the 1970s, serious doubts were being raised about the psychological reality of the abstract rules and structures posited by formal linguistics (see, e.g., Fodor et al., 1974; Halle et al., 1978), and with the exception of a temporary reunion between formal linguistics and psycholinguistics during the 1980s, the two fields largely drifted apart (Ferreira, 2005). While we no doubt still have much to learn about how the brain processes language, many psycholinguists today tend to view formal grammars—at least as those grammars tend to be conceived of by orthodox generative (i.e., Chomskyan) linguists—as poor models of the actual neural goings-on involved in the production and interpretation of language.

Supposing this attitude is right, the question is where this leaves the above account of linguistic structure, which includes hierarchical structure. One might think that, at bottom, formal grammars would appear to be no more than optional technical devices for representing human language sentences. However, it then looks like whether some sentence has this or that hierarchical structure—or indeed, whether it has hierarchical structure *at all*—is similarly a matter of mere representational taste, as suggested by Frank et al. (2012) in a skeptical review of the importance of hierarchical structure in language processing. Could that really be all hierarchical structure in language amounts to? Moreover, how does this square with the fact that the notion of hierarchical structure seems to do genuine explanatory work for us in, e.g., explaining Kanzi’s dramatic dip in accuracy for requests involving NP coordination?

Perhaps some account of linguistic structure anchored in the notion of a formal grammar might ultimately be made to work (particularly if the relevant formal grammar has been formulated with an eye toward better respecting psycholinguistic data^12^). However, I take the foregoing to offer sufficient motivation to at least consider an alternative account that makes a clean conceptual break with formal grammars, and which is instead directly guided by cognitive neuroscience. The next section presents one such account. Put simply, on this account, facts about hierarchical structure in language reduce to facts about how language users mentally represent those sentences. Hierarchical structure in language is inherited from hierarchical cognition.

A final word before getting started. The general idea that there is an evolutionarily significant connection between structure in the linguistic domain and structure in the action domain has been proposed before (see, e.g., Greenfield et al., 1972; Calvin, 1983; Greenfield, 1991; Steedman, 2002; Camps and Uriagereka, 2006; Jackendoff, 2007; Ambrose, 2010; Di Sciullo et al., 2010; Stout, 2011; Boeckx and Fujita, 2014), and hence the particular version of this idea developed here has important theoretical precursors and relatives. Unfortunately, however, given what else I am to accomplish in this article, a discussion of the specific ways in which these various accounts are similar and different would take us too far afield (though see footnote 14 below). Suffice to say that the present account is both less committal on certain points of linguistic detail than some other accounts (and hence compatible with a broader range of conceptions of language in general, and linguistic structure in particular), while simultaneously being more committal on certain points about the hominin technological record, and what that record plausibly tells us about the evolution of relevant human cognitive capacities (e.g., the evolution of our capacity for hierarchical action analysis).

## 2. Humans’ hierarchical action processor

Intuitively, our actions are generally internally structured. Take the act of brushing one’s teeth, for example. This act has parts: picking up one’s toothbrush, applying toothpaste to it, wetting the tip of the brush, followed, of course, by the act of moving one’s brush back and forth over one’s teeth. Just as intuitively, even such pedestrian acts contain subacts. The act of applying toothpaste to one’s toothbrush, for example, can be further decomposed into such acts as picking up the tube of toothpaste, removing its lid, bringing the tube’s opening to the brush’s tip, and squeezing the tube. Actions, too, appear to have nested part-whole structure.

A structured conception of action is natural for a few reasons. For one, in performing complex actions, even ones we are entirely familiar with, we sometimes forget to perform a crucial act component; I may, for example, forget to remove the lid from the tube of toothpaste before attempting to squeeze some onto my brush. Additionally, actions often show what we might call *subact flexibility*: one subact can be swapped for another, without disturbing the other components of the sequence. Suppose, for example, that one of my hands is incapacitated. Then I might instead use my teeth to remove the lid from the toothpaste tube, etc. These and other familiar features of everyday actions certainly make it intuitive to understand actions as being internally structured. For we should not expect such behavior patterns if actions were instead organized as monolithic blocks. However, an analogous question to the one raised about linguistic structure above also arises here, namely: what makes some particular subsequence of physical movements—and *not* certain other subsequences—count as a genuine subact?

It’s commonplace in cognitive neuroscience to likewise see our actions in this way, as sequentially and hierarchically structured “act strings”; what answer does it provide to this question? Arguably the most common answer is that some physical part of an act sequence (e.g., picking up one’s toothbrush) counts as a genuine subact just if the agent has a mentally represented *goal* corresponding to that part (see, in particular, Badre and D’Esposito, 2009; Badre et al., 2009; Christoff et al., 2009).^13^^,^^14^ These goals can be more or less abstract along two dimensions. One is a temporal dimension: a goal can be causally active (i.e., in control of real-time behavior) for a longer or shorter duration. This morning, my goal to get ready for work was in the causal driver’s seat for longer than my goal to brush my teeth. The longer a goal is causally active, the greater its degree of *temporal abstraction* is said to be. The other is a command or control dimension. A goal can be in control of a larger or smaller set of subgoals. My goal to brush my teeth was in fact a subgoal of my goal to get ready for work, and in this sense, the latter was in control of the former. But my goal to get ready for work was also superordinate to a range of other subgoals, such as to shower, eat breakfast, and get dressed. The more subgoals a given goal controls, the greater its degree of *policy abstraction* is said to be. Often, the two kinds of abstraction go together—goals that are more abstract in a policy sense (i.e., that command more goals) also tend to be more abstract in the temporal sense (i.e., active for longer)—though this is not always the case. For example, the goal corresponding to the actual brushing movements I make while brushing my teeth shows considerable temporal abstraction (i.e., it is active for some time), but shows very little (if any) policy abstraction.^15^

Taken at face value, this way of thinking about the organization and control of action implies the existence of a computational system, housed in our brains, wherein the posited goal representations and computational processes are realized. We can think of this system as having something like the following architecture (the exact specs are not crucial for the argument of this article):

- The system contains a multi-layered spatial array that is used as a computational workspace. Representations can be located on the same or different levels in this array. Representations on the same level have a certain linear order with respect to one another. Representations that occur at different levels but stand in a control relation to one another also stand in a hierarchical relation to one another (being either subordinate or superordinate).
- The first step in constructing an action plan is that some high-level goal *G* becomes active. *G* then constrains the selection of subgoals. Specifically, goals, the sequential completion of which is expected to satisfy *G*, are selected. This process repeats, with subgoals triggering the selection of sub-subgoals, etc. All of the goals are written to the layered array such that each higher-level goal controls the lower-level goals whose ordered completion will satisfy it (i.e., the higher-level goal).
- Similarly, the way other representations gain causal efficacy in the system is by becoming *active*, and each goal in the array is either active or inactive at a given time. The activation of a particular goal causes the activation of the first goal it controls. This process continues until a basic level goal is reached. This goal is activated, and once achieved, it is deactivated. Activation then spreads horizontally to the next goal that is controlled by the immediately superordinate goal. Once all of the subgoals controlled by a superordinate goal have been achieved, the superordinate goal is deactivated, and activation then spreads horizontally to the next goal that is on the same level as the superordinate goal.
- If, as sometimes happens, a lower-level goal cannot be achieved, the superordinate goal controlling it remains active while a substitute subgoal is sought. If no alternative subgoal, or sequence of subgoals, can be identified, a substitute for the superordinate goal is sought. The logic behind this design feature is that the system seeks to change the fewest number of goals that will yield a functional action plan. What the system does *not* do is start the entire process of act assembly over from scratch simply because something has gone awry.

**Figure 3:**
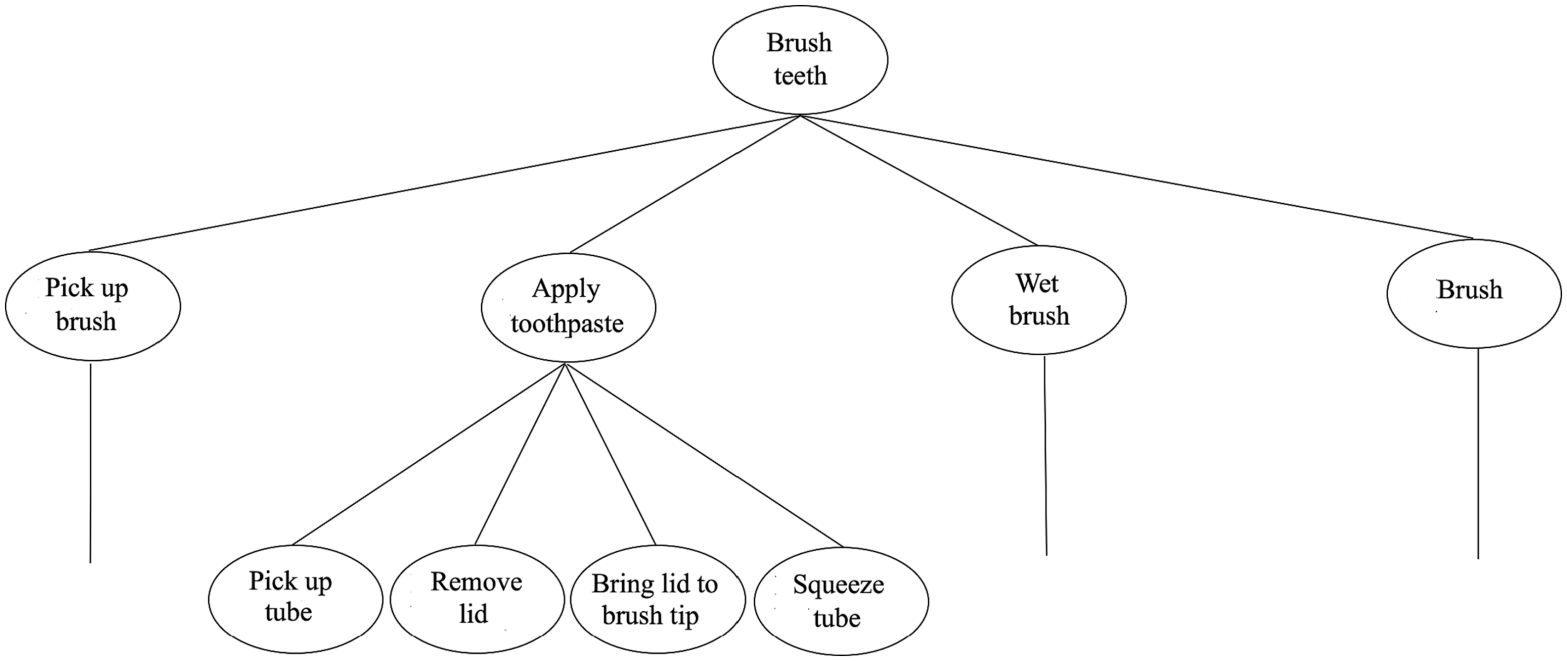
Human’s hierarchical action processor. The figure zooms in on just a single subgoal in the overall sequence, namely, *Apply toothpaste*. Note that some of the subgoals under this goal would likely command still further subgoals (e.g., *Pick up tube* would command the goal *Form a precision grip*, etc.).

A schematic representation of this system is shown in Figure 3. Crucially, there is indeed neuroscientific support for the existence of such a computational system, or something like it. A variety of neuroimaging techniques point to a crucial role for prefrontal cortical areas in realizing the posited goal representations and computational processes. Broca’s area (Brodmann area 45), in particular, has seemed crucial. Distinctive spiking patterns are observed in this area when goals first become active and again when they are completed. Thus, it’s been proposed that Broca’s area plays a role in activating and deactivating goal representations (Koechlin and Jubault, 2006). Moreover, the whole of the prefrontal area appears to be laid out in a gradient fashion, with less abstract representations realized in more caudal (rear) areas, and more abstract representations realized in more rostral (frontal) areas (Badre and D’Esposito, 2009; Dixon et al., 2014). Such neural organization can be understood as the instantiation of the different (functional) levels of the posited spatial array which is used to implement control relations among goal representations.

Moreover, we have had considerable neuroscientific evidence for some time that we make use of much of the same computational machinery in interpreting others’ actions as we do in generating our own actions; we observe highly similar activation patterns in prefrontal areas across both tasks (see, e.g., Decety et al., 1997; Gerardin et al., 2000; Grèzes et al., 2003 Hamzei et al., 2003 Binkofski and Buccino, 2004; Buccino et al., 2004). This fits well with the idea that we (often) explain and predict others’ behavior via *simulation*, that is, using our own mind as a stand-in for others’ (Gallese and Goldman, 1998; Goldman, 2006). This evidence suggests that we somehow run the above system in reverse to arrive at a hierarchically structured representation of a perceived action plan, e.g., we begin by identifying the basic goals underlying another’s act string; from there, the system might initiate a search for a superordinate goal that would be satisfied by the ordered completion of these lower-level goals, etc. In this way, an act observer might go on to eventually attribute quite an abstract goal to the actor.

Finally, it’s also plausible that other primates—or at least Old World Monkeys—share a version of this computational system with us, albeit in a much attenuated form. Premotor cortex area F5 in macaques has been shown to contain neurons which are preferentially activated by the perception of specific action types, such as tearing, grasping, and eating, but also by acts of communication (Di Pellegrino et al., 1992; Gallese et al., 1996; Rizzolatti et al., 1996; Ferrari et al., 2003). Many of these same neurons preferentially fire when the corresponding forms of motor activity are carried out by an agent; for this reason, these neurons were labeled “mirror neurons” (Gallese et al., 1996). Area F5 in macaques is thought to be homologous with Broca’s area in humans. In addition, these neurons in macaques have a cross-modal response profile; they are activated by both visual and auditory signatures of such act types. Broca’s area in humans is similarly cross-modal; it is recruited by tasks as diverse as tool manufacture, music making and listening, and arithmetical calculation (Fadiga et al., 2009). The thread uniting these otherwise disparate tasks is that they all involve the manipulation of hierarchically structured representations. For these reasons, Broca’s area has been described as a “supramodal hierarchical processor” (Fadiga et al., 2009).

## 3. Language structure redux

In this section and the next, I advance two core propositions. First, the above action processor was significantly scaled up over the course of hominin evolution; our ancestors came to possess a version of this system that was far more powerful than anything the other great apes possess. Second, among the things this system was used for was *communication*. It may have been coopted for use in communication only at some later evolutionary stage, or (more likely^16^) it might have always been involved in the control and recognition of communication. Either way, I contend that factors other than communicative ones played a crucial role in driving the hypothesized upgrades to this computational system, especially early on. In this section, I flesh out the account of language structure that emerges on this view. Then I turn to the question of evolutionary drivers.

Some will likely balk at the idea that a computational system designed for the control and recognition of action might be centrally involved in communication. This is understandable; language is clearly a very special thing. Natural languages are extremely complex, highly organized and adapted systems of (generally) arbitrary cultural units. They run on countless culturally evolved conventions, many of which are entirely opaque to language users. These features of language can disguise from us the basic fact that speaking is, at bottom, still a form of complex intentional action. We “do things with words,” as Austin (1962) famously pointed out. But critically, this conception of language is backed by the neuroscience: Broca’s area and other nearby areas—paradigmatic language centers of the brain—all turn out to be multi-modal. Evidently, it’s of no concern to these areas whether the representations they traffic correspond to elements of tool making, music making, or language making; such areas are activated by any form of complex intentional action. None of this is to deny that our brains have been specifically adapted for linguistic communication^17^ over the course of evolutionary history, mind you; almost certainly that’s the case. Language makes cognitive and perceptual demands on users for which it’s well-nigh impossible to identify analogous demands outside the communicative domain. Particularly impressive is the immense speed at which we engage in conversation (Levinson, 2006, 2020; Levinson, n.d.). Conversational responses are typically launched within a 200 msec window of one’s partner concluding her turn (De Ruiter et al., 2006; Levinson and Torreira, 2015; Riest et al., 2015); wait any longer, and one’s silence tends to be interpreted as meaningful (e.g., as signaling disagreement). Moreover, many of the words we so rapidly and fluently use in conversation differ one from another in only the most minute of physical ways (e.g., by individual phonemes). And yet, at the same time, our recognitional capacities for words are robust over a striking range of physical variation. A token of the word “cat” whispered by a little girl and a token of the word “cat” sung by an operatic bassist share almost nothing in common physically, and yet we effortlessly recognize both as tokens of the same type (Dennett, 2017). Very likely, our brain machinery for language has been adapted toward these and other ends. However, all of this is consistent with the basic idea that the brain uses a largely overlapping set of computational resources for both language and action.

With this conceptual hurdle out of the way, let me to now make explicit the account of linguistic structure on offer. The main idea is simple: sentence structure is inherited from the structure of the mental representations underlying sentences. In producing a sentence, a speaker will (we assume) have a particular mental representation of that sentence realized in their action processor. And in this way, the speaker confers a certain structure on the sentence they utter. This includes any nested part-whole structure that might inhere in their mental representation. And similarly, upon interpreting a sentence, a receiver will (we assume) likewise construct a mental representation of the perceived sentence in their action processor, and in this way, confer their *own* structure upon the sentence.

Thus, on this account, facts about the structure of sentences reduce to facts about how senders and receivers mentally represent those sentences. Such an account of structure is clearly *agent-relative*: the same sentence can exhibit different forms of structure for different agents, or even for the same agent at different times (most obviously, at different developmental stages). This is surely a feature rather than a bug, however. In constructing the representations of sentences that they do, senders and receivers will of course be strongly influenced by local, culturally evolved communicative conventions which they have learned. But even where senders and receivers share much knowledge of these conventions in common, their representations can nonetheless diverge in significant respects. For example, it’s plausible that a receiver’s analysis of a sentence is affected by the (perceived) context, and the *stakes* of the context (this is probably true of action interpretation, in general). There are cognitive costs inherent in analyzing a perceived utterance into a complex set of higher- and lower-level linguistic parts, and receivers likely strive to minimize such costs. However, sometimes getting communication *right* can really matter, and then we expect for receivers to bring their full arsenal of interpretative resources to bear. More generally, recognizing this agent relativity of linguistic structure drives home the extent to which it is a genuine *achievement* for senders and receivers to converge on the same or a similar enough representation of a sentence both at and over time in order for communication to succeed.^18^ If, in attempting to communicate their message, the sender makes crucial use of a convention that gives a role to hierarchical relations among the sentence’s parts, then the receiver, too, must obviously attribute hierarchical structure to the sentence in order for communication to reliably succeed. To the extent that the receiver is incapable, finds it particularly challenging, or is simply disinclined to do so, we should expect a communication breakdown in such cases (such as the breakdown we observe when requests featuring NP coordination are made of Kanzi).

One might wonder at this point how the proposed account of linguistic structure actually differs from one logically based on the notion of a formal grammar. There are two main differences, in my view. First, the present account is essentially non-committal on what a speaker’s “knowledge of language” amounts to. It’s equally at home with a wide range of more detailed psycholinguistic models of language processing. Indeed, it could even fit with an explication of a speaker’s knowledge of language based on a particular formal grammar, though in that case the present account might seem redundant. The account’s only real constraint is that our brains house *bona fide* linguistic representations that stand in various structural relations to one another. This is an empirical bet that I think the vast majority of formal linguists, psycholinguists, and cognitive scientists alike would be happy to make. Second, the present account neatly fits, and is indeed directly motivated by, the cognitive neuroscientific evidence. It is a “bottom-up” approach to linguistic structure, and as such, there is little to no scope for a worrying mismatch between linguistic theory and cognitive neuroscientific theory.

At the same time, the present account obviously resists a strongly deflationist attitude regarding hierarchical structure in language. By “strongly deflationist attitude,” I have in mind the idea that hierarchical representations of sentences do not serve to pick out psychologically real forms of structure. Instead, hierarchical representations are seen simply as appealing but ultimately optional ways of publicly representing sentences for the purposes of theoretical discussion (Frank et al., 2012). On this sort of account, it’s natural to doubt that a capacity for working with hierarchically structured signs marks any significant divide in cognitive architecture between humans and other animals (e.g., other great apes). There’s no form of cognition—hierarchical cognition—which we are good at and they are bad at.

In contrast, on the present account, what we instead have are two sets of facts: one set, consisting in how language users in fact mentally represent sentences, and hence the structure they attribute to those sentences; and another set, consisting in how theorists—standing *outside* communicative interactions—represent language users’ sentences, and hence the structure their theoretical representations attribute to sentences. One thing a theorist may wish to do is provide a representation of the actual structure a language user attributes to a sentence. If so, then the theorist gets things right to the extent that these two sets of facts match up. But often theorists will not get things right (not least because how language users mentally represent structure can be expected to vary at and over time). However, sometimes a theorist might be interested in something other than how things are psychologically with a given language user. Then they might posit and work with a model of structure which they find useful for other reasons (e.g., economy). In any case, the point is that it’s crucial to distinguish these two sets of facts, lest we mistake theory for reality. To drive home this point, consider the following proposal made by Levinson (2013). He suggests that center embedding first evolved in the context of conversation (granted, conversation structure is different from sentence structure, but I think the example is still apt). Levinson takes off from the observation that many speakers struggle to parse sentences exhibiting even minimal degrees of multiple center embedded constituents, such as (5):

(5) The boy the dog the cat scratched bit went to hospital.

Levinson then points out that, in stark contrast, we generally find it effortless to cope with multiple center embedded structures at the discourse level. Take (6):

(6) 1. A: May I have a coffee, please?
 2. B: Which kind would you like?
 3. A: Which kind do you have?
 4. B: We have black coffee, cappuccinos, lattes …
 5. A: I’ll have a latte, please.
 6. B: Sure thing, coming right up.

(6) is composed of three question-answer pairs, with the third pair embedded inside the second pair which is embedded inside the first pair. Hence, (5) and (6) show an equal degree of (iterative) center embedding. However, whereas (5) is already very hard for speakers of English to process, we have no problem at all engaging in the bit of dialog shown in (6). Indeed, as Levinson points out, it’s entirely commonplace for conversation to exemplify many additional levels of center embedding.

Let us grant that representing conversation in this way is useful/illuminating for some theoretical purpose. The point is this: it is clearly a further question whether the conversational participants’ representation of the conversation features center embedding. Here, I shall remain neutral on this question other than to say it’s not obvious that they must do so in order for conversation to run smoothly.^19^ Perhaps it is enough, computationally speaking, for each participant to simply keep a running list of the questions that have been asked so far, and which of these questions has already been answered. All they then need to do is tailor their response at each step in the conversation to the last unanswered question. *Prima facie*, anyway, it seems possible that these task demands might be managed computationally without the need to token a mental representation that is center embedded. But either way, the bottom line is this: how we as theorist-onlookers represent language is one thing; how those engaged in actual linguistic communication represent things is another.

Let me finish out this section by returning to the basic issue of evidence for hierarchical structure in language. On the present account, language users’ intuitions are still crucial. But now note that the intuitive judgment that, e.g., “the boy with the broken leg” is a part of (1) can indeed be understood as causally driven by a psychologically real form of structure; users have this intuition because they represent these words as forming a cohesive constituent or chunk of the sentence. But in addition, the to-and-fro of conversation arguably provides persuasive evidence that language users’ frequently attribute nested part-whole structure to sentences. Specifically, I have in mind here patterns of other- and self-initiated repair. Repair initiators have been shown to occur on average once per every 80 s of human linguistic conversation (Dingemanse et al., 2015; Albert, 2018; Micklos and Woensdregt, 2022). They range from the very general (e.g., “huh?”) to the very specific (e.g., “which boy was it that missed the game?”). A robust finding is that conversational participants generally try to make the task of repair as easy as possible for their interlocutors by providing as specific a repair initiator as they can (Levinson, n.d.) (you do not simply say “huh?” if you have grasped that your conversational partner is trying to tell you something about a person who missed the game). This plausibly reflects the extent to which participants view conversation as a cooperative (and norm-governed) enterprise (Enfield, 2017; Enfield and Sidnell, 2022). Much can be gleaned from repair initiators about how language users represent linguistic structure in real time. Consider these:

(7) Which boy missed the game?
(8) Who missed the game?

Imagine that both (7) and (8) are repair initiators uttered in response to (1) (i.e., “The boy with the broken leg missed the game.”). (7) is more specific than (8), but both are perfectly natural (i.e., we can easily imagine circumstances in which each would be the most natural response). Each repair initiator provides a window on how the interpreter has represented (1). An interpreter would not utter (7) unless he represented (1) as having the structure, “the boy *X* missed the game,” where *X* is a placeholder for a constituent. What (7) in effect asks his interlocutor is how he should fill in this placeholder. Similarly, an interpreter would not utter (8) unless he represented (1) as having the structure, “*X* missed the game.” Again: what (8) in effect asks is how this placeholder should be filled in. (7) thus provides evidence that the interpreter represents the language following “the boy” and preceding “missed the game” as forming cohesive chunk, while (8) provides evidence that the interpreter represents the language preceding “missed the game” as a cohesive chunk. It’s also easy to see how a tendency to produce repair initiators like (7) and (8) might also function to *induce* new ways of representing sentence constituents among language users over time. In this way, the back-and-forth of conversation can catalyze and/or further encourage the representation of various forms of linguistic structure.

## 4. Technique and technology as evolutionary drivers

With this account of linguistic structure under our belts, let us now turn to the issue of how the computational machinery supporting such structure arose and stabilized over the course of evolutionary time? What drove these evolutionary changes in our cognitive architecture? Selection pressures created by hominin tool manufacture were very likely crucial.

All of us great apes are extractive foragers; we seek out and feed on a variety of encased foods. Toward this end, we use our mouths and hands, but also tools of various kinds. This fact has shaped not just our bodies, but also our minds. That said, human ways of making and using tools are obviously quite special. We make vastly more tools, many of which are vastly more complex. Moreover, we have literally come to depend for our survival on our tools in a way that no other great ape does; we are, you might say, obligate technophiles. This has probably been true of hominins for at least a million and half years (Shea, 2017). Natural selection has thus had ample time to mold our minds for more complex and efficient tool use and manufacture. Almost certainly, this has included significantly upgraded capacities of hierarchical cognition.

Our great ape cousins use stone as a technological resource, but also organic materials such as wood, and the same was no doubt true of our earliest ancestors. However, organic tools are very unlikely to endure over hundreds of thousands of years, much less for over a million. So, in attempting to understand the deep history of hominin technology and its cognitive demands, it is the lithic record on which we must ultimately depend.^20^ For present purposes, the following rather coarse grained breakdown will suffice.

### 4.1. Oldowan

The earliest stone tools belong to an industry called the *Oldowan*. The very earliest such tools are now estimated to date to 3.3 mya (Harmand et al., 2015), though there is evidence of cut-marked bones somewhat earlier (McPherron et al., 2010). Oldowan tools were extremely primitive; it would not be too much of an exaggeration to say that all one needed to do to make such a tool is hit two stones (of the right sort) together. And while variation in the size and shape of tools is visible both between and within Oldowan assemblages, it is widely held that such variation is fully explicable in terms of differences among the raw materials that were worked, noise inherent in the production process, and gross anatomical features (e.g., hand morphology) of Oldowan tool makers. What we do *not* see is any signal of directional, incremental change. The character of these assemblages suggests that tool makers did not intend to impose a particular form on their tools beyond producing a useable cutting edge. Lithic technology remained at this very primitive stage of development for well over a million years. Accordingly, it’s unlikely that the production of Oldowan signals a cognitive upgrade in our line. Indeed, it has been shown that orangutans can spontaneously produce such tools if provided with the right raw materials and context (Motes-Rodrigo et al., 2022).

### 4.2. Acheulean

Around 1.8 mya we see the appearance of a novel technological industry epitomized by the Acheulean handaxe. Very likely, the development of these new tools was spurred on by the increasing role of meat (and hence butchering) in hominin diets (Toth and Schick, 2019). Whereas it can often take a careful and experienced eye to recognize an Oldowan tool *as* a tool, the handaxes and other large cutting tools of the Acheulean are unmistakable pieces of hominin technology. Many handaxes, particularly as we move forwards in time, were extensively bifacially flaked and symmetrical along multiple dimensions.^21^ Their production very likely required enhanced forms of executive control, planning, and (spatial) reasoning. In addition to having a mental template of the finished artifact form, handaxe makers almost certainly implemented complex actions plans which could be adjusted on the fly in response to unexpected outcomes (e.g., the detachment of a much larger or smaller flake than one intended, or perhaps no flake removal at all) (Hiscock, 2014; Planer, 2017). The handaxe form was famously stable over vast stretches of time and space. Debate continues over why this was, and what it tells us about the social learning abilities of Acheulean age hominins (more on this below). However, on virtually everybody’s view, the Acheulean industry minimally signals a significant upgrade in individual cognitive abilities for act organization and control.

### 4.3. Levallois and composite tools

As we enter the Middle Pleistocene (770 kya – 126 kya) we encounter two striking developments: Levallois and composite tools. Levallois tools are famous for the very elaborate degree of platform preparation they involve: removing certain flakes so as to be in a position to remove certain other flakes, etc. A core is worked and reworked by the knapper until it is finally possible for them to extract the fully formed Levallois flake tool from the core with a single, final blow (Kuhn, 2020). Many humans find Levallois tools extremely visually attractive, as did their makers no doubt. But more to the present point: the extensive preparatory flaking sequences necessitated by Levallois tool making strongly suggests highly complex and flexible action plans.

The same can be said for composite tools, the other main tool-related innovation we know of from this period. The paradigm composite tool features a stone blade of some kind fixed to a handle with some form of adhesive (e.g., a birch resin gum). If the skilled manufacture of handaxes and Levallois tools is demanding of significant technical and natural history knowledge (and it is), then that is true in spades for composite tools. The manufacture of adhesives that required careful heat treatment is particularly impressive. The production of a composite tool was a complex, multi-step process requiring the seamless integration of semantic and procedural knowledge from a variety of domains (Barham, 2013). Over and above a sophisticated action plan, composite tools arguably indicate a significant increase in what Steven Mithen (1996) has called “cognitive fluidity.”

There is obviously much more that can be said at this point, but the foregoing is already adequate for our purposes. A number of theorists have posited a connection between stone tool manufacture, increased cognitive capacities, and language.^22^ I uphold that trend here. But my claim is a rather specific one, namely: as tool making and use became increasingly complex and central to our ancestors’ lifeways, our hierarchical action processor was significantly scaled up. It was hominin technique and technology which, at least initially, drove major upgrades to this computational system.^23^ Below are several ways that a precursor version of this system present in the last common ancestor with *Pan* might have been incrementally enhanced.

- *Activity duration*. Recall that goal representations gain causal efficacy though becoming activated. One way the system can go from less to more powerful is by being able to maintain its goals, including higher-level ones, in an active state for longer. The period of time a superordinate goal can be kept in an active state has consequences for the number of subgoals (as well as the temporal abstractness of subgoals) it can realistically command. All other things being equal, a capacity to maintain activation patterns for longer should allow for the construction of more complex action sequences. This is likely to involve the inhibition of competing goals (distractors), and hence upgraded executive control.
- *Breadth of planning*. Generally speaking, more complex action plans feature a larger number of goals per level of the spatial array. Thus, the system might evolve to stably hold more goal representations per row. In effect, this would allow for action plans exhibiting a greater degree of *horizontal complexity*.
- *Depth of planning*. This feature directly relates to the system’s capacity for assembling hierarchically structured mental representations. The system can go from having fewer to more such levels at which (representations of) act components can be stored and executed. As a result, action plans exhibiting greater *vertical complexity* become possible.
- *State transitions.* Once each of the subgoals controlled by a superordinate goal have been achieved, the superordinate goal is deactivated and activation spreads to the next goal at the same level. Fast, fluent action requires that this process happen swiftly and seamlessly. This is clearly something which evolution and/or learning can tinker with. Most obviously, the system can evolve so that these transitions among goal states happen more rapidly.
- *Search efficiency.* Once a superordinate goal has become active, a sequence of subgoals the serial execution of which can be expected to achieve the superordinate goal must be identified. This task can obviously be carried out more or less efficiently. Similar remarks apply in the case where the system is unable to achieve a particular subgoal and hence must search for a replacement subgoal or superordinate goal.

As communication in our line became more complex, it’s not only possible but likely that this system underwent additional evolution, making it better suited to handling specifically linguistic tasks. For example, as noted above, normal conversation demands that we make our conversational contributions approximately within a 200 msec window of each other. Computationally speaking, this means things have to work fast. Selective pressures emanating from the communicative domain might have been crucial in ramping up the computational speed of this system. But, I suggest, these were likely to be add-ons to a system that had already grown much more computationally complex in response to tool-related selective pressures.

## 5. Hierarchical cognition in other great apes

Allow me to summarize the argument so far. We began by noting that while hierarchical structure in language appears to be a genuine thing, and appears to do genuine explanatory work for us, the standard way of thinking about such structure (i.e., in terms of being generated by a formal grammar of some kind) does not mesh well with our neurobiological understanding of language. This motivated our search for an alternative account of linguistic structure with more neurobiological credibility. I argued that an account of structure based in humans’ hierarchical action processor provides just this and has other attractive features. Finally, I suggested that it was selection for enhanced technology-related capacities that likely drove the early elaboration of this computational system in our line.

**Figure 4:**
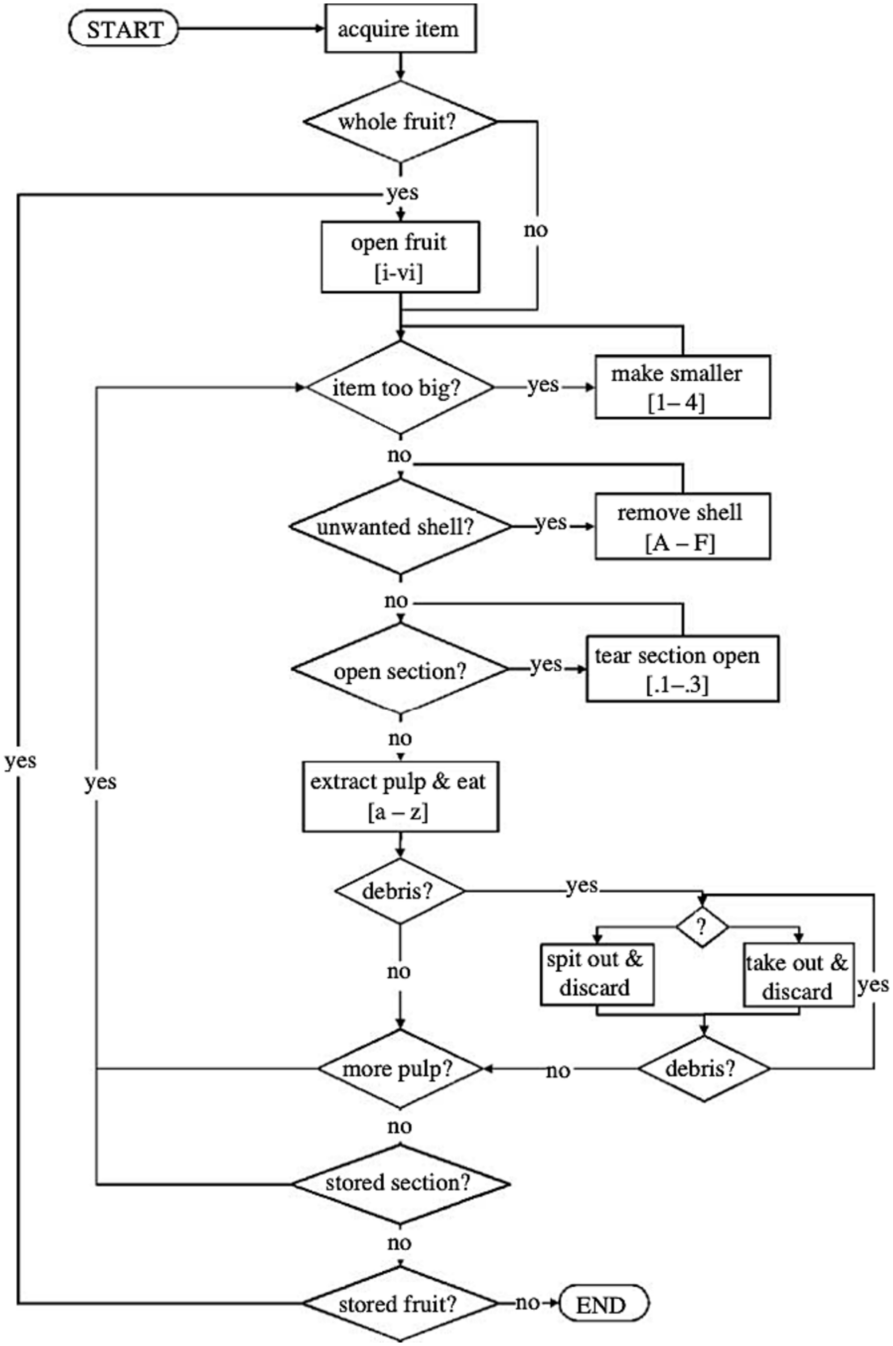
A decision-making tree for chimpanzee Saba fruit processing. Taken from Byrne (2007). Reproduced with permission (Royal Society).

There is a puzzle, however, which the reader may have already spotted. The puzzle is this. To the extent that, say, brushing one’s teeth is generated by a hierarchical action plan, then it must be that the other great apes are capable of far from trivial forms of hierarchical cognition. For they engage in a number of behaviors that rival the act of brushing one’s teeth in terms of internal complexity. The most obvious examples that come to mind are behaviors such as nut cracking (Boesch and Boesch, 1983), termite fishing (Goodall, 1963), honey extraction (Boesch and Boesch, 1990), and nest building (Goodall, 1962)—all acquired behaviors, mind you—but there are likely many, many more, particularly if we are willing to include behaviors of enculturated great apes. Consider the flow chart from Byrne (2007), which is intended to depict the decisions made by a chimpanzee as it consumes *Saba* fruit (Figure 4). In describing chimpanzees’ behavior in such terms, Byrne is guided by the same sorts of considerations about subact omission, flexibility, repeatability, etc., that motivate the idea that brushing one’s teeth and other similar actions are genuinely hierarchically structured. While Byrne’s conventions for representing act structure are clearly different, it’s easy to see how the structure he posits might be reinterpreted in the framework that was set out above. What we would wind up with is an action plan that is very clearly hierarchically structured.

However, if this is so, then why is it that even very rudimentary forms of hierarchically structured communication have not evolved in the other great ape lines? Or even more puzzlingly: why is it than even highly enculturated great apes like Kanzi cannot learn to fluently process quite basic hierarchically structured sentence representations? *Pan* non-communicative behavior plausibly exemplifies at least as many levels of nested part-whole structure as is needed to process this sentence. What gives? Clearly, the account as presented so far cannot be a complete story.

Two hypotheses suggest themselves. One is that hierarchical cognition involving representations of *meaningful* behaviors (e.g., words) is more challenging for some reason than hierarchical cognition that does not (i.e., which is limited “merely” to practical, non-communicative behaviors, such as picking up a stick/toothbrush). The idea would then be that, while other great apes are capable of hierarchical representation outside the communicative domain, the semantic dimension of the acts and subacts involved in communication causes some form of processing difficulty for them. We might call this the *meanings-are-hard hypothesis*. While I think this is a possibility, it is hard to see any evidence directly in support of it. Here I want to explore an alternative though ultimately compatible line of thought which makes more contact with empirical evidence.

In Planer and Sterelny (2021), we in effect assumed that selection for enhanced capacities for hierarchical cognition in actors would automatically result in enhanced capacities for hierarchically analyzing others’ action strings. Thus, as the sophistication of individual action plans increased, so too did the sophistication of others’ understanding of the corresponding actions (so the thinking went). The guiding idea here was that in humans, but also in other primates to some extent, brain areas that are active during act performance are also active during act perception (see the discussion toward the end of Section 2 above).

I now think this is likely too fast. And note: if the ability to implement hierarchical action plans in one’s own behavior indeed comes apart from the ability to reliably recognize hierarchical structure in others’ actions, then we have a solution to the above puzzle.^24^ It seems undeniable that great ape forms of behavior such as nut cracking, termite fishing, and the like imply hierarchical action plans. But the evolution of the ability to hierarchically *analyze* others’ behavior (nevermind to do so accurately and rapidly) may well have required significant downstream evolution.

We might call this the *analyses-are-hard hypothesis*. Unfortunately, not much is known about the ability of great apes to perceive hierarchical structure in others’ action strings. Some work was done in this area in the late 1990s/early 2000s (Byrne, 1998; Byrne and Russon, 1998; Whiten, 2000; Whiten, 2002) and it was claimed at that time that wild great apes can not only copy sequentially structured action plans, but also hierarchically structured ones (implying an ability to hierarchically analyze others’ action streams). A much discussed case was that of nettle leaf processing in gorillas (i.e., the particular way in which gorillas fold nettle leaves so as to make their consumption safe). However, a lot has changed in social learning theory since that time. In particular, many putative cases of know-how copying among wild great apes have turned out to be more plausibly understood as involving transmission via non-copying social learning mechanisms.^25^ As regards the specific case of gorilla nettle processing, for example: it was later shown that naïve gorillas spontaneously innovate similar processing methods on their own, calling into question the idea that the behavior is transmitted via know-how copying among wild gorillas (Tennie et al., 2008). To conclusively demonstrate that unenculturated great apes copy others’ hierarchical action plans, it’s necessary to show that the corresponding form of action does not fall within the relevant ape species’ *zone of latent solutions* (roughly, the set of behaviors an average member of the relevant species can realistically innovate on their own, given appropriate social and environmental scaffolding) (Tennie et al., 2009, 2020a).^26^ If the form *does* lie within this zone (and there is evidence of social learning) then the reappearance of behavioral forms across actors should rather be understood in terms of the social transmission of other types of knowledge (e.g., know-where, know-what, etc.), or else in terms of a process of know-how triggering^27^ (Bandini et al., 2020). The mere fact (if it is one) that one ape comes to harbor a hierarchical action plan of his own that resembles the hierarchical action plan of a model does not in itself demonstrate copying of hierarchical structure. Unfortunately, studies that would allow us to reach firmer conclusions on such questions simply have not been carried out yet.^28^

That having been said, one might think there is good indirect evidence that other great apes lack the ability to copy hierarchically structured action plans (or at the very least, that such an ability is much attenuated in them compared to us). In particular, one might think that the hominin technological record strongly suggests that this ability did not evolve in our line until long after we split from the *Pan* lineage. In such a case, the most parsimonious conclusion would be that the ability to copy hierarchically structured action plans is a derived hominin trait. I briefly unpack this line of thinking in the next and final section.

## 6. The evolution of hierarchical action analysis and copying

On the framework set out in this article, the ability to perceive hierarchical structure in others’ action goes hand in hand with the ability to copy said structure, and so, archeological signatures of the latter constitute plausible archeological signatures of the former. Why the tight connection here? When we interpret another’s act stream in hierarchical terms, we attribute to them an action plan consisting in a set of sequentially and hierarchically organized goal representations. As noted above, this in effect amounts to running one’s own action system in reverse. What we do is perceive certain bodily movements, which we then attempt to subsume under certain low-level goals. In turn, we then attempt to subsume these goals under higher-level, superordinate goals, etc. Assuming all goes smoothly, one’s own action processor comes to house an action plan that matches that underlying the observed behavior. To reproduce the observed hierarchical behavior, then, all one must do is execute this action plan.

So, once one has recovered the action plan underlying another’s behavior, copying the corresponding behavior is trivial. However, the ability to analyze an action stream at multiple hierarchical levels in the first place is likely far from trivial. Probably the easiest way to see this is to note that act analysis poses a kind of binding problem which is altogether absent in the case of act performance: in analyzing an action, not only must one distinguish signal (i.e., intentional bodily movement) from noise; in addition, one must frame various hypotheses about which subacts work together as an organized subplan for achieving certain superordinate goals. This can easily lead to a combinatorial explosion in the absence of specialized biases and heuristics. Moreover, act analysis often imposes significant memory demands. In attempting to decipher the goal underlying a particular beahvior, we often rely on the behavior’s outcome, together with cues produced by the agent (does the agent look pleased with the outcome?) I can infer that some novel behavior of yours was intended to, e.g., open the pickle jar, if the jar in fact opens and you appear to be satisfied with this outcome. But as act sequences come to be longer and more complex—as they come to exhibit greater breadth and depth—the rationale for various subacts, especially those coming early on in the sequence, may well remain hidden until the observed act nears or reaches completion. I may only be in a position to recognize an extended sequence of small flake removals as (e.g.) motivated by the goal of thinning out the handaxe once I see you remove a long thin flake from across the entire face of the core at the end of this sequence. Unless I am able to keep sensory representations of your earlier subacts active in my mind during the meantime, I will be unable to recover the structure of your action plan.

But to get back to the main thread of the argument: everyone agrees that humans possess the ability to copy others’ hierarchically organized actions, and hence that this ability evolved at some point in our evolutionary past. The question is *when*. Here, the hominin lithics record provides invaluable data.

There is no reason to invoke the ability to copy hierarchical structure in order to explain Oldowan tools. As mentioned, while it is true that there is variation over time and space in Oldowan assemblages, there is no signal of cumulative, directional change in Oldowan technique or technology. Oldowan tools are extremely primitive and can in fact be reinnovated on the spot by both other great apes as well modern humans with no previous knowledge of stone tools.^29^ Crucially, this is *not* to say that social learning played no role in Oldowan tool making; on the contrary, it likely did. In particular, tool makers likely learned which raw materials to use to manufacture tools in part by observing one another. However, such social learning is quite distinct from copying others hierarchically structured action strings. It is social learning of a rather low-key sort that a wide variety of animals plausibly exhibit (van Shaik, 2010).

What about the Acheulean? What is uncontroversial—or at least what *should* be uncontroversial, in my view—is that the manufacture of Acheulean grade tools demanded a non-trivial upgrade in hominins’ hierarchical cognitive capacities. Acheulean tool makers almost certainly needed enhanced abilities for planning, controlling, and flexibly organizing action in real time. Among other things, this would explain why not even enculturated great apes can be trained to make handaxes. However, it is still a further question whether such tools provide compelling evidence for hierarchical copying abilities. The main evidence in support of such a scenario^30^ has always been the striking degree of *stability* we see in handaxe form over vast stretches of time and space. To make a handaxe is no trivial feat—it requires genuine skill—and yet hominins all over the Old World, and for a period of well over a million years, managed to reliably reproduce this form. Surely, this implies hierarchical copying abilities, right?

Skeptics flip this intuitive argument on its head. For starters, the signature handaxe form appears in hominin populations that were apparently culturally isolated from one another. In particular, handaxes have been discovered from sites in China where the nearest handaxe possessing population was located over 1,500 km away (Zhang et al., 2010; Corbey et al., 2016). Such a geographical distribution strongly argues for local reinnovation of handaxe design, as opposed to cultural transmission via copying. But even more tellingly: such stasis in handaxe design is the opposite of the pattern we would expect if toolmakers were in fact copying each other’s tool manufacture technique (Foley, 1987; Richerson and Boyd, 2008; Tennie et al., 2017; Tennie, 2023). Famously, by accumulating the modifications of multiple (generations of) learners, copying social learning tends to rapidly produce cultural variants whose complexity and/or arbitrariness put them well beyond what any individual could realistically innovate on his or her own in a single lifetime. At the same time, we do not expect the same modifications to accumulate in informationally isolated populations; that would be a miracle. Social learners will add to pre-existing forms differently, and often in ways that have far reaching consequences for future design possibilities. Thus, all other things being equal, we expect copying social learning to produce locally distinctive cultural traditions in populations lacking informational contact.

There is not scope to provide a full defense for such a non-copying scenario here;^31^ it will have to suffice to say that I think such a scenario is very much a live option, particularly as regards the first half of the Acheulean (i.e., > 1.2 mya). On this view, the lithic evidence suggests that hierarchical copying abilities likely did not evolve in our line until the late Early Pleistocene/early Middle Pleistocene. Shortly thereafter, we see the appearance of new and even more demanding tool forms (Levallois, composite), as well as the emergence of distinctive local traditions, plausibly implicating hierarchical copying. If something along the lines of this view is correct, then the total set of facts about how human hierarchical cognition compares to that of the other great apes looks something like this:

- Humans have evolved enhanced means of *organizing and controlling action*. As tool manufacture and use became more sophisticated in our line, and as these skills became increasingly central to our lifeways, our action control system was adapted in ways that enabled more complexly structured forms of behavior. This included, but was almost certainly not limited to, increased planning depth.^32^ The complexity inherent in manufacturing handaxes strongly suggests that such adaptations had established in our line by the time our ancestors were routinely manufacturing Acheulean grade tools.
- But humans *also* evolved enhanced capacities for *action analysis*, and more specifically, for recognizing the hierarchical actions plans behind others’ complex action streams. In line with my remarks at the start of this section, this presumably required the evolution of an additional suite of cognitive adaptations, as act interpretation poses computational problems that are absent in the case of act performance.^33^ As the Middle Pleistocene got underway, tool manufacture and use was becoming even more complex in our line. Eventually (we can assume), hominin technological skills passed a complexity threshold which selected for the ability to copy others’ hierarchical action plans. It began to *pay* to reliably discern this structure. Hominins with more and better skills for copying such plans would have acquired their groups’ demanding technical skill set more rapidly and hence more cheaply (i.e., they would have incurred fewer learning and opportunity costs). Such copying abilities required, and in turn delivered, enhanced capacities for hierarchical analysis of others’ action.

Both sets of facts are critical to a full understanding of the evolution of hierarchically structured communication as we now know it in humans. As senders, we repeatedly nest linguistic constituents inside of other linguistic constituents, etc., building up complex sentences that exhibit many levels of hierarchical structure. Moreover, we do so with great speed and agility. This reflects our nature as highly skilled organizers and controllers of action [as per (i)]. And as receivers, we routinely recover the hierarchical structure of others’ utterances accurately, or at least accurately enough, for communication to in general succeed—also with tremendous speed and flexibility. This reflects our nature as highly skilled analysts of others’ actions, of which others’ utterances are a special case [as per (ii)].

However, it is specifically (ii) that provides an answer to the puzzle raised in the last section. More precisely: the reason, I contend, that only humans evolved hierarchically structured communication is that only humans—under selection pressure for the ability to copy others’ action plans—evolved the ability to reliably recognize the hierarchical structure inherent in others’ action strings. Only we became expert analysts of the internal structure of others’ actions. Once we did, the stage was set for the cultural emergence of communicative conventions that exploited hierarchical relations among sentence parts to assign meaning to whole sentences.^34^ With both senders and receivers attributing similar forms of hierarchical structure to utterance strings, such structure could come to play a central role in our communication via well-understood processes of sender and receiver co-adaptation to one another’s communicative policies (Planer and Godfrey-Smith, 2021). In other words: nested part-whole structure could begin to evolve, presumably in the service of increased economy, into the communicative staple we know it to be in human languages today.

## Data availability statement

The original contributions presented in the study are included in the article/supplementary material, further inquiries can be directed to the corresponding author.

## Author contributions

The author confirms being the sole contributor of this work and has approved it for publication.

## Conflict of interest

The author declares that the research was conducted in the absence of any commercial or financial relationships that could be construed as a potential conflict of interest.

## Publisher’s note

All claims expressed in this article are solely those of the authors and do not necessarily represent those of their affiliated organizations, or those of the publisher, the editors and the reviewers. Any product that may be evaluated in this article, or claim that may be made by its manufacturer, is not guaranteed or endorsed by the publisher.

## Footnotes

^1^It is understandable why, as a certain type of grammar is required to generate both types of structure. But more on this below.

^2^Interestingly, as pointed out by Levinson (2013), arguments similar to Everett’s had in fact already been made many years prior by Ken Hale on the basis of certain Australian Aboriginal languages (Hale, 1976).

^3^There is a striking paucity of plausible candidates from animal communication studies of signals whose parts work together to mean something over and above the whole (Scott-Phillips, 2015). The main case that has been discussed in the literature is the “pyow-hack” signal of putty-nosed monkeys. Suffice it to say that the jury is still out on whether this signal functions as an idiomatic expression or one in which the meaning of the parts, together with their mode of composition, function to determine a meaning that goes beyond the meaning of the parts alone. See Schlenker et al. (2016) for a good overview of different interpretations of the pyow-hack signal. Planer and Godfrey-Smith (2021) advance a general framework which recognizes more types of structured signals than is common which is useful for thinking about the pyow-hack cases and other similar ones.

^4^See Savage-Rumbaugh et al. (1993).

^5^Truswell (2017) provides a detailed discussion of both the criteria and reasons for discarding certain cases from his sample.

^6^Not least because many of the other sentences in the corpus required Kanzi to be sensitive to the order of word items, i.e., the sentence’s linear structure.

^7^See, also, Hurford (2011) for a similar diagnosis of Kanzi’s linguistic difficulties.

^8^For a fuller discussion, see Planer and Sterelny (2021), Ch. 5.

^9^Non-terminal symbols correspond to the categories of the language (e.g., NP, VP, etc.). Terminal symbols correspond to linguistic items that can appear in a completed sentence (e.g., “the,” “boy,” etc.). Below, I use capital and lower case letters to signify non-terminal and terminal symbols, respectively.

^10^It’s easy to see that center embedding requires a particular kind of rule of this kind, e.g., A ➔ BAC. This rule can be used to generate a string with an arbitrary number of center embeddings: BAC, BBACC, BBBACCC, and so on.

^11^See, e.g., Davis et al. (1994) for more on these formal themes. And see, also, Planer and Sterelny (2021), Ch. 5, for a less formal treatment of the same.

^12^Combinatory categorical grammar (see, e.g., Ades and Steedman, 1982; Steedman, 1996, 2000) would be an example of such a formal grammar.

^13^An alternative view holds that it is *instrumental relations* holding among the various physical parts of an action that confer upon some of those parts their status as genuine subacts (see, e.g., Botvinick, 2008). The idea is that some parts of the action are causal prerequisites for others. Structural facts map to these causal facts. So, picking up one’s toothbrush counts as a subact because it *sets up* or *makes possible* the act of brushing. This proposal is preferred by those who wish to remain neutral on how (if at all) actions are mentally represented by agents. It is attractive to theorists of a behaviorist stripe.

^14^There is also a line of thought among some cognitive neuroscientists of action, and some language evolution theorists interested in the cognitive neuroscience of action, that in effect attempts to answer the what-counts-as-a-genuine-subact question by again appealing to the notion of a formal grammar (see, e.g., Jackendoff, 2007; Stout, 2011; Boeckx and Fujita, 2014). More specifically, the idea is that actions have the structure they do in virtue of being generated by a formal “action grammar.” As will become clear below, I share with these theorists the goal of providing a unified account of linguistic and action structure. Unsurprisingly, however, I am skeptical that the way to go is to understand action as generated by a formal grammar. As in the case of language, unless action grammars are psychologically real entities which agents use to generate and understand actions, it’s unclear what the notion of action structure actually amounts to. Is it merely a theoretical convenience to think of actions as being internally structured, or does this correspond to something real on the part of actions? Worse yet, one might think it is fundamentally misguided to understand action as being generated by a formal grammar. A grammar generates some strings and *not* others. The strings it does not generate are *ill-formed* or *ungrammatical* by the lights of the grammar. It is far from clear that it even makes sense to think of (non-communicative) actions in this way, as capable of being ill-formed or ungrammatical. It is of course true that there are many actions that are not *physically* possible, but that does not seem like the same thing.

^15^In what follows, it’s the notion of policy abstraction that’s most relevant, and talk of abstraction should be taken in this sense unless otherwise specified. For it is policy abstraction that is directly related to the construction of mental representations with hierarchical (i.e., nested part-whole) structure.

^16^As the evidence of F5 neurons in macaques tuned to communicative acts arguably suggests.

^17^And likely for communication as such (i.e., whether or not that communication involves language).

^18^See Dor (2015) for a similar idea (he conceives of senders and receivers as having to overcome an “experiential gap”).

^19^Though if they did (need to), I would claim that the present action processor account of structure is well poised to explain how they do so. There is no need to think of the account as limited solely to the case of sentence structure.

^20^For some excellent work on the cognitive demands of different lithic industries, see Muller et al. (2017) and Muller et al. (2022). See, also, earlier work by Stout and Chaminade (2009, 2012) and Stout (2011).

^21^The term *developed Oldowan* is often used to describe the more refined tool specimens of the late Acheulean (i.e., from around 1 mya – 0.8 mya).

^22^Some more recent examples include: Stout and Chaminade (2009, 2012), Davidson (2010), Stout (2011), Lombao et al. (2017), Planer (2017), Planer and Sterelny (2021), and Pain (2023).

^23^I say “at least initially” here, as I think other forms of complex intentional action which our ancestors engaged in, such as music, dance, and art, likely served as additional drivers of upgrades to this system. For each of these activities are clearly highly complex forms of intentional action, too (they are also paradigmatically arbitrary cultural forms, hence forms that cannot be realistically rediscovered by individuals). However, the archeological evidence suggests that these activities were largely or entirely late Pleistocene innovations (Conard, 2005, 2007; Henshilwood, 2014).

^24^Though there may be other solutions, too. Also, note that the *meanings-are-hard* and the *analyses-are-hard* hypotheses are not incompatible. I shall leave consideration of a hybrid of the two hypotheses for another day, however.

^25^See Motes-Rodrigo and Tennie (2021) for an up-to-date summary.

^26^See, also Tomasello (1996) for a critical precursor of the notion of a species’ zone of latent solutions. Put simply, what Tomasello suggested at that time was that rather than copying others’ behaviors, unenculturated chimpanzees instead learn about environmental affordances from watching others’ behaviors. They then discover for themselves the same or similar behaviors targeting these affordances.

^27^As when one person’s yawn causes another person to yawn. See Sperber (2000), Tennie (2023), Buskell and Tennie (n.d.), and Planer et al. (Forthcoming) for a discussion of triggering vs. copying proper.

^28^A recent chapter by Tennie et al. (2020b) urges the need for precisely such research.

^29^See Motes-Rodrigo et al. (2022) on apes; Snyder et al. (2022) on humans.

^30^Though not always under the present description.

^31^See Planer et al. (Forthcoming) for a more extended defense.

^32^Recall the discussion towards the end of Section 4: activity duration, planning breadth, state transitions, and search efficiency are all other areas in which incremental improvements might have occurred.

^33^In this article, I have focused on the cognitive dimension of analyzing others’ actions. But there is, of course, an emotional-motivational dimension to this activity as well (Tomasello, 1999). (I thank a referee for reminding me of this.) Individuals must in fact have the social motivation, not just the cognitive wherewithal, to do so. It is likely that increased, and increasingly complex, joint action as practiced by our ancestors played an important role in ratcheting up this motivation (Tomasello, 2010; Tomasello et al., 2012). I see such a suggestion as complementary to the current proposal (not least because tool manufacture—especially in its more complex varieties—was likely a joint social activity at times).

^34^An example from Berwick and Chomsky (2016) provides a simple illustration of such a convention. Take the sentence, “Intuitively, birds that fly swim.” Berwick and Chomsky note that English speakers interpret “intuitively” in this sentence as modifying the meaning of “swim” not of “fly.” This is so despite the fact that “fly” is closer in terms of linear distance to “intuitively” than it is to “swim.” We can nicely explain (and arguably cannot explain otherwise) this fact about English speakers by assuming that (a) they represent the sentence hierarchically, and (b) follow a convention on which it is the hierarchical, not linear, distance between the adverb heading the sentence and the sentence’s other constituents which determines what the adverb modifies (and hence what the sentence as a whole means). More specifically, “intuitively” is understood as modifying “swim” rather than “fly” because “intuitively” is separated by fewer hierarchical levels (less hierarchical distance) from “swim” than it is from “fly.” Such a convention uses hierarchical relations among sentence constituents to determine sentence meaning.

## References

[ref1] Ades A. E. Steedman M. J. (1982). On the order of words. Linguist. Philos. 4, 517–558. doi: 10.1007/BF00360804

[ref2] Albert S., & Ruiter, J.P. de. (2018). Repair: the Interface between interaction and cognition. Top. Cogn. Sci., 10, 279–313, doi: 10.1111/tops.12339, PMID: 29749039 PMC6849777

[ref3] Ambrose S. H. (2010). Coevolution of composite-tool technology, constructive memory, and language: implications for the evolution of modern human behavior. Curr. Anthropol. 51, S135–S147. doi: 10.1086/650296

[ref4] Austin J. (1962). How to do things with words. Oxford: Oxford University Press.

[ref5] Badre D. D’Esposito M. (2009). Is the rostro-caudal Axis of the frontal lobe hierarchical? Nat. Rev. Neurosci. 10, 659–669. doi: 10.1038/nrn2667, PMID: 19672274 PMC3258028

[ref6] Badre D. Hoffman J. Cooney J. W. D'Esposito M. (2009). Hierarchical cognitive control deficits follow- ing damage to the human frontal lobe. Nat. Neurosci. 12, 515–522. doi: 10.1038/nn.2277, PMID: 19252496 PMC2990342

[ref7] Bandini E. Motes-Rodrigo A. Steele M. P. Rutz C. Tennie C. (2020). Examining the mechanisms underlying the acquisition of animal tool behaviour. Biol. Lett. 16:20200122. doi: 10.1098/rsbl.2020.0122, PMID: 32486940 PMC7336849

[ref8] Barham L. (2013). From hand to handle: The first industrial revolution. New York: Oxford University Press.

[ref9] Berwick R. C. Chomsky N. (2016). Why only us: Language and evolution. MIT press, Cambridge, MA.

[ref10] Binkofski F. Buccino G. (2004). Motor functions of the Broca’s region. Brain Lang. 89, 362–369. doi: 10.1016/S0093-934X(03)00358-4 15068919

[ref11] Boeckx C. A. Fujita K. (2014). Syntax, action, comparative cognitive science, and Darwinian thinking. Front. Psychol. 5:627. doi: 10.3389/fpsyg.2014.00627 25018738 PMC4073197

[ref12] Boesch C. Boesch H. (1983). Optimisation of nut-cracking with natural hammers by wild chimpanzees. Behaviour. 83, 265–286. doi: 10.1163/156853983X00192

[ref13] Boesch C. Boesch H. (1990). Tool use and tool making in wild chimpanzees. Folia Primatol. 54, 86–99. doi: 10.1159/000156428, PMID: 2157651

[ref14] Botvinick M. M. (2008). Hierarchical models of behavior and prefrontal function. Trends Cogn. Sci. 12, 201–208. doi: 10.1016/j.tics.2008.02.009, PMID: 18420448 PMC2957875

[ref15] Buccino G. Lui F. Canessa N. Patteri I. Lagravinese G. Benuzzi F.. (2004). Neural circuits involved in the recognition of actions performed by nonconspecifics: an fMRI study. J. Cogn. Neurosci. 16, 114–126. doi: 10.1162/089892904322755601, PMID: 15006041

[ref16] Buskell A. Tennie C. (Forthcoming). Mere recurrence and cumulative culture at the margins. Br. J. Philos. Sci.

[ref17] Byrne R. W. (1998). “Of priming and program-level copying” in Intersubjective communication and emotion in early ontogeny, 228.

[ref18] Byrne R. W. (2007). Culture in great apes: using intricate complexity in feeding skills to trace the evolutionary origin of human technical prowess. Philos. Trans. R. Soc. B Biol. Sci. 362, 577–585. doi: 10.1098/rstb.2006.1996, PMID: 17289650 PMC2346518

[ref19] Byrne R. W. Cartmill E. Genty E. Graham K. E. Hobaiter C. Tanner J. (2017). Great ape gestures: intentional Communi- cation with a rich set of innate signals. Anim. Cogn. 20, 755–769. doi: 10.1007/s10071-017-1096-4, PMID: 28502063 PMC5486474

[ref20] Byrne R. W. Russon A. E. (1998). Learning by imitation: a hierarchical approach. Behav. Brain Sci. 21, 667–684. doi: 10.1017/S0140525X98001745, PMID: 10097023

[ref21] Calvin W. H. (1983). The throwing Madonna: Essays on the brain. McGraw Hill, New York, NY.

[ref22] Camps M. Uriagereka J. (2006). “The Gordian knot of linguistic fossils” in The biolinguistic turn: Issues on language and biology. eds. Roselló J. Martín J. (Barcelona: PPU), 34–65.

[ref23] Carroll S. B. (2005). Endless forms most beautiful: The new science of evo devo and the making of the animal kingdom (No. 54). New York: WW Norton & Company.

[ref24] Charniak E. McDermott D. (1985). Introduction to artificial intelligence. Addison Wesley, Reading, MA.

[ref25] Chomsky N. (1959). On certain formal properties of grammars. Inf. Control. 2, 137–167. doi: 10.1016/S0019-9958(59)90362-6

[ref26] Christoff K. Keramatian K. Gordon A. M. Smith R. Mädler B. (2009). Prefrontal Organization of Cognitive Control According to levels of abstraction. Brain Res. 1286, 94–105. doi: 10.1016/j.brainres.2009.05.096, PMID: 19505444

[ref27] Conard N. J. (2005). “An overview of the patterns of behavioural change in Africa and Eurasia during the middle and late Pleistocene” in From tools to symbols: from early hominids to modern humans. Johannesburg: Blackwell, Wits University Press 294–332.

[ref28] Conard N. (2007). “Cultural evolution in Africa and Eurasia during the middle and late Pleistocene” in Handbook of paleoanthropology. eds. Henke W. Tattersall I. (Berlin: Springer), 2001–2037.

[ref29] Corbey R. Jagich A. Vaesen K. Collard M. (2016). The acheulean handaxe: more like a bird's song than a beatles' tune? Evol. Anthropol. Issues News Rev. 25, 6–19. doi: 10.1002/evan.21467, PMID: 26800014 PMC5066817

[ref30] Davidson I. (2010). “Stone tools and the evolution of hominin and human cognition” in Stone tools and the evolution of human cognition. Boulder: University of Colorado Press. 185–205.

[ref31] Davis M. Sigal R. Weyuker E. J. (1994). Computability, complexity, and languages: Fundamentals of theoretical computer science. San Diego: Academic Press.

[ref32] De Ruiter J. P. Mitterer H. Enfield N. J. (2006). Projecting the end of a speaker's turn: a cognitive cornerstone of conversation. Language 82, 515–535. doi: 10.1353/lan.2006.0130

[ref33] Decety J. Grezes J. Costes N. Perani D. Jeannerod M. Procyk E.. (1997). Brain activity during observation of actions. Influ- ence of action content and subject’s strategy. Brain a. J. Neurol. 120, 1763–1777. 10.1093/brain/120.10.1763 9365369

[ref34] Dennett D. C. (2017). From bacteria to Bach and back: The evolution of minds. New York: WW Norton & Company.

[ref35] Di Pellegrino G. Fadiga L. Fogassi L. Gallese V. Rizzolatti G. (1992). Understanding motor events: a neurophysiological study. Exp. Brain Res. 91, 176–180. doi: 10.1007/BF00230027 1301372

[ref36] Di Sciullo A. M. Piattelli-Palmarini M. Wexler K. Berwick R. C. Boeckx C. Jenkins L.. (2010). The biological nature of human language. Biolinguistics 4, 4–34. doi: 10.5964/bioling.8759

[ref37] Dingemanse M. Roberts S. G. Baranova J. Blythe J. Drew P. Floyd S.. (2015). Universal principles in the repair of communication problems. PLoS One 10:e0136100. doi: 10.1371/journal.pone.0136100, PMID: 26375483 PMC4573759

[ref38] Dixon M. L. Fox K. C. Christoff K. (2014). Evidence for rostro-caudal functional Organization in Multiple Brain Areas Related to goal-directed behavior. Brain Res. 1572, 26–39. doi: 10.1016/j.brainres.2014.05.012, PMID: 24842002

[ref39] Dor D. (2015). The instruction of imagination: Language as a social communication technology. Foundations of Human Interaction. Oxford University Press, New York, NY.

[ref40] Enfield N. J. (2017). How we talk: The inner workings of conversation. New York: Basic Books.

[ref41] Enfield N. J. Sidnell J. (2022). Consequences of language: From primary to enhanced intersubjectivity. Cambridge, MA: The MIT Press, Cambridge, MA.

[ref42] Everett D. (2005). Cultural constraints on grammar and cognition in Pirahã: another look at the design features of human language. Curr. Anthropol. 46, 621–646. doi: 10.1086/431525

[ref43] Fadiga L. Craighero L. D’Ausilio A. (2009). Broca’s area in language, action, and music. Ann. N. Y. Acad. Sci. 1169, 448–458. doi: 10.1111/j.1749-6632.2009.04582.x, PMID: 19673823

[ref1001] Ferreira F. (2005). Psycholinguistics, formal grammars, and cognitive science Linguist. Rev. 22, 365–380.

[ref44] Ferrari P. F. Gallese V. Rizzolatti G. Fogassi L. (2003). Mirror neurons responding to the Observa- tion of Ingestive and communicative mouth actions in the monkey ventral premotor cortex. Eur. J. Neurosci. 17, 1703–1714. doi: 10.1046/j.1460-9568.2003.02601.x, PMID: 12752388

[ref1002] Fitch W. T. Hauser M. D. (2004). Computational constraints on syntactic processing in a nonhuman primate. Science 303, 377–380. 14726592 10.1126/science.1089401

[ref45] Fodor J. Bever A. Garrett T. G. (1974). The psychology of language: An introduction to psycholinguistics and generative grammar. New York: McGraw-Hill.

[ref46] Foley R. (1987). Hominid species and stone-tool assemblages: how are they related? Antiquity 61, 380–392. doi: 10.1017/S0003598X00072938

[ref47] Frank S. L. Bod R. Christiansen M. H. (2012). How hierarchical is language use? Proc. R. Soc. B Biol. Sci. 279, 4522–4531. doi: 10.1098/rspb.2012.1741, PMID: 22977157 PMC3479729

[ref48] Gallese V. Fadiga L. Fogassi L. Rizzolatti G. (1996). Action recognition in the premotor cortex. Brain 119, 593–609. doi: 10.1093/brain/119.2.593 8800951

[ref49] Gallese V. Goldman A. (1998). Mirror neurons and the simulation theory of mind-reading. Trends Cogn. Sci. 2, 493–501. doi: 10.1016/S1364-6613(98)01262-5, PMID: 21227300

[ref1003] Gentner T. Q. Fenn K. M. Margoliash D. Nusbaum H. C. (2006). Ecursive syntactic pattern learning by songbirds. Nature 440, 1204–1207. 16641998 10.1038/nature04675 PMC2653278

[ref50] Genty E. Byrne R. W. (2010). Why do gorillas make sequences of gestures? Anim. Cogn. 13, 287–301. doi: 10.1007/s10071-009-0266-4, PMID: 19649664

[ref51] Gerardin E. Sirigu A. Lehéricy S. Poline J. B. Gaymard B. Marsault C.. (2000). Partially overlapping neural networks for real and imagined hand movements. Cereb. Cortex 10, 1093–1104. doi: 10.1093/cercor/10.11.1093, PMID: 11053230

[ref52] Goldman A. I. (2006). Simulating minds: The philosophy, psychology, and neuroscience of mindreading. New York: Oxford University Press.

[ref53] Goodall J. M. (1962). Nest building behavior in the free ranging chimpanzee. Ann. N. Y. Acad. Sci. 102, 455–467. doi: 10.1111/j.1749-6632.1962.tb13652.x, PMID: 13949060

[ref54] Goodall J. (1963). “The feeding behaviour ofwild chimpanzees: a preliminary report” in Symp. Zool. Soc. Lond, vol. 10, 39–47.

[ref55] Greenfield P. M. (1991). Language, tools and brain: the ontogeny and phylogeny of hierarchically organized sequential behavior. Behav. Brain Sci. 14, 531–551. doi: 10.1017/S0140525X00071235

[ref56] Greenfield P. M. Nelson K. Saltzman E. (1972). The development of rulebound strategies for manipulating seriated cups: a parallel between action and grammar. Cogn. Psychol. 3, 291–310. doi: 10.1016/0010-0285(72)90009-6

[ref57] Grèzes J. Armony J. L. Rowe J. Passingham R. E. (2003). Activations related to “mirror” and “canonical” neurones in the human brain: an fMRI study. NeuroImage 18, 928–937. doi: 10.1016/S1053-8119(03)00042-9, PMID: 12725768

[ref58] Haegeman L. (1991). Introduction to government and binding theory. Wiley-Blackwell, Hoboken, NJ.

[ref59] Hale K. (1976). “The adjoined relative clause in Australia” in Grammatical categories in Australian languages. New Jersey: Humanities Press vol. 78, 105.

[ref60] Halle M. Bresnan J. Miller G. A. (1978). Linguistic theory and psychological reality. MIT Press, Cambridge, MA.

[ref61] Hamzei F. Rijntjes M. Dettmers C. Glauche V. Weiller C. Büchel C. (2003). The human action recognition system and its relationship to Broca’s area: an fMRI study. NeuroImage 19, 637–644. doi: 10.1016/S1053-8119(03)00087-9, PMID: 12880794

[ref62] Harmand S. Lewis J. E. Feibel C. S. Lepre C. J. Prat S. Lenoble A.. (2015). 3.3-million-year-old stone tools from Lomekwi 3, West Turkana, Kenya. Nature 521, 310–315. doi: 10.1038/nature14464, PMID: 25993961

[ref1004] Hauser M. D. Chomsky N. Fitch W. T. (2002).The faculty of language: what is it, who has it, and how did it evolve? Science 298, 1569–1579. 12446899 10.1126/science.298.5598.1569

[ref63] Henshilwood C. (2014). Origins of symbolic behavior. In McGraw-hill yearbook of Science & Technology. California: McGraw-Hill.

[ref64] Herbert S. (1962). The architecture of complexity. Proc. Am. Philos. Soc. 106, 467–482.

[ref65] Hiscock P. (2014). Learning in lithic landscapes: a reconsideration of the hominid “toolmaking” niche. Biol. Theory 9, 27–41. doi: 10.1007/s13752-013-0158-3

[ref66] Hurford J. (2011). The origins of grammar: Language in the light of evolution II. Oxford: Oxford University Press.

[ref67] Jackendoff R. (2007). Language, consciousness, culture: Essays on mental structure. Cambridge, MA: MIT Press.

[ref68] Koechlin E. Jubault T. (2006). Broca’s area and the hierarchical organization of human behavior. Neuron 50, 963–974. doi: 10.1016/j.neuron.2006.05.017, PMID: 16772176

[ref69] Kuhn S. L. (2020). The evolution of Paleolithic technologies. London: Routledge.

[ref70] Levinson S. (2006). Cognition at the heart of human interaction. Discourse Stud. 8, 85–93. doi: 10.1177/1461445606059557

[ref71] Levinson S. C. (2013). Recursion in pragmatics. Language 89, 149–162. doi: 10.1353/lan.2013.0005

[ref72] Levinson S. C. (2020). “On the human “interaction engine”” in Roots of human sociality. eds. Enfield N. J. Levinson S. C. (New York: Routledge), 39–69.

[ref73] Levinson S. C. (Forthcoming). The Interaction Engine and the Evolution of Language.

[ref74] Levinson S. C. Torreira F. (2015). Timing in turn-taking and its implications for processing models of language. Front. Psychol. 6:731. doi: 10.3389/fpsyg.2015.00731 26124727 PMC4464110

[ref75] Lombao D. Guardiola M. Mosquera M. (2017). Teaching to make stone tools: new experimental evidence supporting a technological hypothesis for the origins of language. Sci. Rep. 7:14394. doi: 10.1038/s41598-017-14322-y, PMID: 29089534 PMC5663762

[ref1005] Makuuchi M. Bahlmann J. Anwander A. Friederici A. D. (2009). Segregating the core computational faculty of human language from working memory. Proc. Natl. Acad. Sci. 106, 8362–8367. 19416819 10.1073/pnas.0810928106 PMC2688876

[ref76] McPherron S. P. Alemseged Z. Marean C. W. Wynn J. G. Reed D. Geraads D.. (2010). Evidence for stone-tool-assisted consumption of animal tissues before 3.39 million years ago at Dikika, Ethiopia. Nature 466, 857–860. doi: 10.1038/nature09248, PMID: 20703305

[ref77] Micklos A. Woensdregt M. (2022). Cognitive and Interactive Mechanisms for Mutual Understanding in Conversation. PsyArXiv. doi: 10.31234/osf.io/aqtfb

[ref78] Mithen S. (1996). The prehistory of the mind. London: Phoenix Books.

[ref79] Motes-Rodrigo A. McPherron S. P. Archer W. Hernandez-Aguilar R. A. Tennie C. (2022). Experimental investigation of orangutans’ lithic percussive and sharp stone tool behaviours. PLoS One 17:e0263343. doi: 10.1371/journal.pone.0263343, PMID: 35171926 PMC8849460

[ref80] Motes-Rodrigo A. Tennie C. (2021). The method of local restriction: in search of potential great ape culture-dependent forms. Biol. Rev. 96, 1441–1461. doi: 10.1111/brv.12710, PMID: 33779036

[ref81] Muller A. Clarkson C. Shipton C. (2017). Measuring behavioural and cognitive complexity in lithic technology throughout human evolution. J. Anthropol. Archaeol. 48, 166–180. doi: 10.1016/j.jaa.2017.07.006

[ref82] Muller A. Shipton C. Clarkson C. (2022). Stone toolmaking difficulty and the evolution of hominin technological skills. Sci. Rep. 12:5883. doi: 10.1038/s41598-022-09914-2, PMID: 35393496 PMC8989887

[ref83] Nevins A. Pesetsky D. Rodrigues C. (2009). Pirahã exceptionality: a reassessment. Language 85, 355–404. doi: 10.1353/lan.0.0107

[ref84] Pain R. (2023). Stone tools, predictive processing and the evolution of language Mind & Language. 38, 711–731.

[ref1006] Planer R. J. Bandini E. Tennie C. (Forthcoming). Hominin Tool Evolution and Its (Surprising) Relation to Language Origins. Oxford Handbook of Approaches to Language Evolution. New York: Oxford University Press.

[ref85] Planer R. J. (2014). Replacement of the “genetic program” program. Biol. Philos. 29, 33–53. doi: 10.1007/s10539-013-9388-9

[ref86] Planer R. J. (2017). Talking about tools: did early Pleistocene hominins have a protolanguage? Biol. Theory 12, 211–221. doi: 10.1007/s13752-017-0279-1

[ref87] Planer R. J. Godfrey-Smith P. (2021). Communication and representation understood as sender–receiver coordination. Mind Lang. 36, 750–770. doi: 10.1111/mila.12293

[ref88] Planer R. Sterelny K. (2021). From signal to symbol: The evolution of language. MIT Press, Cambridge, MA.

[ref89] Richerson P. J. Boyd R. (2008). Not by genes alone: How culture transformed human evolution. Chicago: University of Chicago Press.

[ref90] Riest C. Jorschick A. B. de Ruiter J. P. (2015). Anticipation in turn-taking: mechanisms and information sources. Front. Psychol. 6:89. doi: 10.3389/fpsyg.2015.00089 25699004 PMC4313610

[ref91] Rizzolatti G. Fadiga L. Gallese V. Fogassi L. (1996). Premotor cortex and the recognition of motor actions. Cogn. Brain Res. 3, 131–141. doi: 10.1016/0926-6410(95)00038-0 8713554

[ref92] Savage-Rumbaugh E. S. Murphy J. Sevcik R. A. Brakke K. E. Williams S. L. Rumbaugh D. M.. (1993). Language comprehension in ape and child. Monogr. Soc. Res. Child Dev. 58, 1–222. doi: 10.2307/1166068, PMID: 8366872

[ref93] Schlenker P. Chemla E. Arnold K. Zuberbühler K. (2016). Pyow-hack revisited: two analyses of putty-nosed monkey alarm calls. Lingua 171, 1–23. doi: 10.1016/j.lingua.2015.10.002

[ref94] Scott-Phillips T. (2015). Speaking our minds. London: Palgrave-Macmillan.

[ref95] Shea J. J. (2017). Occasional, obligatory, and habitual stone tool use in hominin evolution. Evol. Anthropol. Issues News Rev. 26, 200–217. doi: 10.1002/evan.21547, PMID: 29027335

[ref96] Snyder W. D. Reeves J. S. Tennie C. (2022). Early knapping techniques do not necessitate cultural transmission. Sci. Adv. 8:eabo2894. doi: 10.1126/sciadv.abo2894, PMID: 35857472 PMC9258951

[ref97] Sperber D. (2000). “An objection to the memetic approach to culture” in Darwinizing culture: The status of memetics as a science. ed. Aunger R. (New York: Oxford University Press), 163–173.

[ref98] Steedman M.. (1996), Surface structure and interpretation, MIT Press, Cambridge, MA.

[ref99] Steedman M. (2000). The syntactic process. MIT press, Cambridge, MA.

[ref100] Steedman M. (2002). Plans, affordances, and combinatory grammar. Linguist. Philos. 25, 723–753. doi: 10.1023/A:1020820000972

[ref101] Stout D. (2011). Stone toolmaking and the evolution of human culture and cognition. Philos. Trans. R. Soc. B 366, 1050–1059. doi: 10.1098/rstb.2010.0369, PMID: 21357227 PMC3049103

[ref102] Stout D. Chaminade T. (2009). Making tools and making sense: complex, intentional behaviour in human evolution. Camb. Archaeol. J. 19, 85–96. doi: 10.1017/S0959774309000055

[ref103] Stout D. Chaminade T. (2012). Stone tools, language and the brain in human evolution. Philos. Trans. R. Soc. B 367, 75–87. doi: 10.1098/rstb.2011.0099, PMID: 22106428 PMC3223784

[ref104] Tennie C. (2023). The earliest tools and cultures of hominins.

[ref105] Tennie C. Bandini E. Van Schaik C. P. Hopper L. M. (2020a). The zone of latent solutions and its relevance to understanding ape cultures. Biol. Philos. 35, 1–42. doi: 10.1007/s10539-020-09769-9 PMC7548278 33093737

[ref106] Tennie C. Call J. Tomasello M. (2009). Ratcheting up the ratchet: on the evolution of cumulative culture. Philos. Trans. R. Soc. B Biol. Sci. 364, 2405–2415. doi: 10.1098/rstb.2009.0052, PMID: 19620111 PMC2865079

[ref107] Tennie C. Hedwig D. Call J. Tomasello M. (2008). An experimental study of nettle feeding in captive gorillas. Am. J. Primatol. Off. J. Am. Soc. Primatol. 70, 584–593. doi: 10.1002/ajp.20532, PMID: 18330896

[ref108] Tennie C. Hopper L. M. van Schaik C. P. (2020b). “On the origin of cumulative culture: consideration of the role of copying in culture-dependent traits and a reappraisal of the zone of latent solutions hypothesis” in Chimpanzees in context: A comparative perspective on chimpanzee behavior, cognition, conservation, and welfare. eds. Hopper L. M. Ross S. R. (Chicago: University of Chicago Press), 428–453.

[ref109] Tennie C. Premo L. S. Braun D. R. McPherron S. P. (2017). Early stone tools and cultural transmission: resetting the null hypothesis. Curr. Anthropol. 58, 652–672. doi: 10.1086/693846

[ref110] Tomasello M. (1996). “Do apes ape” in Social learning in animals: The roots of culture. San Diego: Academic Press 319–346.

[ref1007] Tomasello M. (1999). The human adaptation for culture. Annu. Rev. Anthropol. 28, 509–259.

[ref111] Tomasello M. (2010). Origins of human communication. MIT press, Cambridge, MA.

[ref112] Tomasello M. Melis A. P. Tennie C. Wyman E. Herrmann E. (2012). Two key steps in the evolution of human cooperation: the interdependence hypothesis. Curr. Anthropol. 53, 673–692. doi: 10.1086/668207

[ref113] Toth N. Schick K. (2019). Why did the Acheulean happen? Experimental studies into the manufacture and function of Acheulean artifacts. Anthropologie 123, 724–768. doi: 10.1016/j.anthro.2017.10.008

[ref114] Truswell R. (2017). Dendrophobia in bonobo comprehension of spoken English. Mind Lang. 32, 395–415. doi: 10.1111/mila.12150

[ref115] van Shaik C. P. (2010). “Social learning and culture in animals” in Animal behaviour: Evolution and mechanisms. Berlin, Heidelberg: Springer 623–653.

[ref116] Whiten A. (2000). Primate culture and social learning. Cogn. Sci. 24, 477–508. doi: 10.1207/s15516709cog2403_6

[ref117] Whiten A. (2002). “Imitation of sequential and hierarchical structure in action: experimental studies with children and chimpanzees” in Imitation in animals and artifacts. Cambridge: MIT Press. 191–209.

[ref118] Zhang P. Huang W. Wang W. (2010). Acheulean handaxes from Fengshudao, Bose sites of South China. Quat. Int. 223-224, 440–443. doi: 10.1016/j.quaint.2009.07.009

